# The Role of the Anti-Aging Protein Klotho in IGF-1 Signaling and Reticular Calcium Leak: Impact on the Chemosensitivity of Dedifferentiated Liposarcomas

**DOI:** 10.3390/cancers10110439

**Published:** 2018-11-14

**Authors:** Vanessa Delcroix, Olivier Mauduit, Nolwenn Tessier, Anaïs Montillaud, Tom Lesluyes, Thomas Ducret, Frédéric Chibon, Fabien Van Coppenolle, Sylvie Ducreux, Pierre Vacher

**Affiliations:** 1 INSERM U1218, F-33076 Bordeaux, France; olivier.mauduit@live.fr (O.M.) anais.montillaud@hotmail.fr (A.M.); tom.lesluyes@inserm.fr (T.L.); frederic.chibon@inserm.fr (F.C.); 2 Department of Life and Health Sciences, University of Bordeaux, F-33000 Bordeaux, France; thomas.ducret@u-bordeaux.fr; 3 Department of Pathology, Bergonié Cancer Institute, F-33076 Bordeaux, France; 4 University of Lyon, F-69361 Lyon CEDEX 07, France; nolwenn.tessier@univ-lyon1.fr (N.T.); fabien.van-coppenolle@univ-lyon1.fr (F.V.C.); sylvie.ducreux@univ-lyon1.fr (S.D.); 5 Univ-Lyon, CarMeN laboratory, Inserm U1060, INRA U1397, Université Claude Bernard Lyon 1, INSA Lyon, Hospital Cardiology, B13 Building, Lyon East, F-69500 Bron, France; 6 INSERM U1045, Centre de Recherche Cardio-Thoracique de Bordeaux (CRCTB), F-33000 Bordeaux, France

**Keywords:** klotho, cancer, liposarcoma, calcium, ER stress, translocon, IGF-1, ERK, gemcitabine, TRPC6, channels

## Abstract

By inhibiting Insulin-Like Growth Factor-1-Receptor (IGF-1R) signaling, Klotho (KL) acts like an aging- and tumor-suppressor. We investigated whether *KL* impacts the aggressiveness of liposarcomas, in which IGF-1R signaling is frequently upregulated. Indeed, we observed that a higher *KL* expression in liposarcomas is associated with a better outcome for patients. Moreover, *KL* is downregulated in dedifferentiated liposarcomas (DDLPS) compared to well-differentiated tumors and adipose tissue. Because DDLPS are high-grade tumors associated with poor prognosis, we examined the potential of KL as a tool for overcoming therapy resistance. First, we confirmed the attenuation of IGF-1-induced calcium (Ca^2+^)-response and Extracellular signal-Regulated Kinase 1/2 (ERK1/2) phosphorylation in *KL*-overexpressing human DDLPS cells. *KL* overexpression also reduced cell proliferation, clonogenicity, and increased apoptosis induced by gemcitabine, thapsigargin, and ABT-737, all of which are counteracted by IGF-1R-dependent signaling and activate Ca^2+^-dependent endoplasmic reticulum (ER) stress. Then, we monitored cell death and cytosolic Ca^2+^-responses and demonstrated that KL increases the reticular Ca^2+^-leakage by maintaining TRPC6 at the ER and opening the translocon. Only the latter is necessary for sensitizing DDLPS cells to reticular stressors. This was associated with ERK1/2 inhibition and could be mimicked with IGF-1R or MEK inhibitors. These observations provide a new therapeutic strategy in the management of DDLPS.

## 1. Introduction

The heterogeneous family of soft-tissue sarcomas (STS) encompasses rare malignant tumors that arise from mesenchymal tissue. The histological and molecular analyses of these tumors allow for the identification of numerous histotypes, including liposarcomas (LPS) that display an adipocytic differentiation and are divided into four histological subtypes: well-differentiated (WDLPS), dedifferentiated (DDLPS), myxoid round cell (MRCLPS), and pleomorphic (PLPS) liposarcomas [1,2]. WDLPS are the most frequent LPS (40% of cases) and are associated with good prognosis. However, in 10% of cases, they can dedifferentiate and give rise to DDLPS that are high-grade tumors. DDLPS mainly occur de novo and account for 25% of all LPS. These tumors are associated with a poor outcome [3] due to metastatic progression (17–30%), high local recurrence rates (41–57%) [4,5] and high chemoresistance. By contrast, MRCLPS are radiosensitive and chemoresponsive. PLPS are highly aggressive but, fortunately, very rare tumors (5% of LPS).

For now, complete surgical resection is the mainstay of therapy for LPS patients. Due to the resistance of these tumors to standard chemotherapies [6], patients suffering from advanced, unresectable, or metastatic LPS receive adriamycin- or gemcitabine-based regimens as palliative treatments. Identifying new therapeutic strategies and response biomarkers is of the utmost importance in order to manage high-grade LPS.

One of the altered pathways that is subject to targeted therapy is IGF-1R signaling. Indeed, IGF-1R is upregulated in osteosarcoma (OS) tumors compared to normal tissue [7] and is associated with a poor overall [8] and metastasis-free survival in cohorts of OS and STS patients [8,9]. Moreover, its downstream effectors p-AKT and p-ERK were suggested as biomarkers for the local recurrence in STS [10]. Both *IGF-1* and *IGF-2* mRNA [11] and IGF-1R [12] are upregulated in LPS tumors compared to adipose tissue. Considering the importance of insulin/IGF-1 signaling in adipose tissue homeostasis, as demonstrated by mice lacking *IGF-1R/IR* (Insulin Receptor) expression [13], and in oncogenesis [14], IGF-1R inhibitors are attractive for LPS therapy. A drug synergistic screen suggested the combined inhibition of IGF-1R and Cyclin-Dependent Kinase 4 (CDK4) as a promising strategy for DDLPS [15]. However, IGF-1R targeted therapies have proved to be disappointing in clinical trials [16], even in OS and STS [17]. So, new strategies for inhibiting insulin/IGF-1 signaling are still needed as a monotherapy or in combination with other drugs to overcome resistance.

*KL (klotho)* was first discovered in mice mutated for this gene: the animals displayed multiple aging-like phenotypes and died prematurely [18]. The overexpression of *kl* in mice increases their lifespan up to 19–30% [19]. *KL* encodes a transmembrane protein whose extracellular domain can be shed by secretases and act as a soluble hormone [20]. The alternative splicing of the gene can also produce a soluble form of the protein (KLs) that will be directly secreted into the extracellular environment [21]. The transmembrane protein is an obligatory co-factor for FGF23 (Fibroblast Growth Factor 23) and so, has a crucial role in the normal renal function and in phosphate and calcium (Ca^2+^) homeostasis [22]. Moreover, KLs and membrane-bound KL exhibit anti-aging properties mainly by inhibiting insulin/IGF-1 signaling and reducing oxidative stress [19]. Interestingly, *kl* knockout in mice results in the absence of adipose tissue [18]. Indeed, Klotho regulates proliferation and adipogenic differentiation of adipose-derived stem cells [23].

Numerous studies have shown that *KL* is frequently downregulated in cancer (gliomas, breast, colorectal, lung cancers, etc.). KL exerts a tumor suppressive effect mainly through inhibition of IGF-1 signaling [24,25]. Considering its role in adipose tissue homeostasis, we examined the clinical relevance of *KL* in terms of the survival of LPS patients and the hypothetical alteration of its expression in these tumors. Then, in a DDLPS cell line, we investigated the effect of *KL* overexpression on IGF-1 signaling and on tumoral phenotypes, especially chemoresistance. Finally, we identified the molecular targets of KL and confirmed the involved mechanisms with two other DDLPS cell lines. Based on our findings, we suggest a new potential therapeutic approach for the management of DDLPS, relying on the disruption of intracellular Ca^2+^ homeostasis combined with endoplasmic reticulum (ER) stressors.

## 2. Results

### 2.1. KL Expression Has a Prognostic Value for Liposarcoma Patients and Is Downregulated in DDLPS Tumors

**Figure 1:**
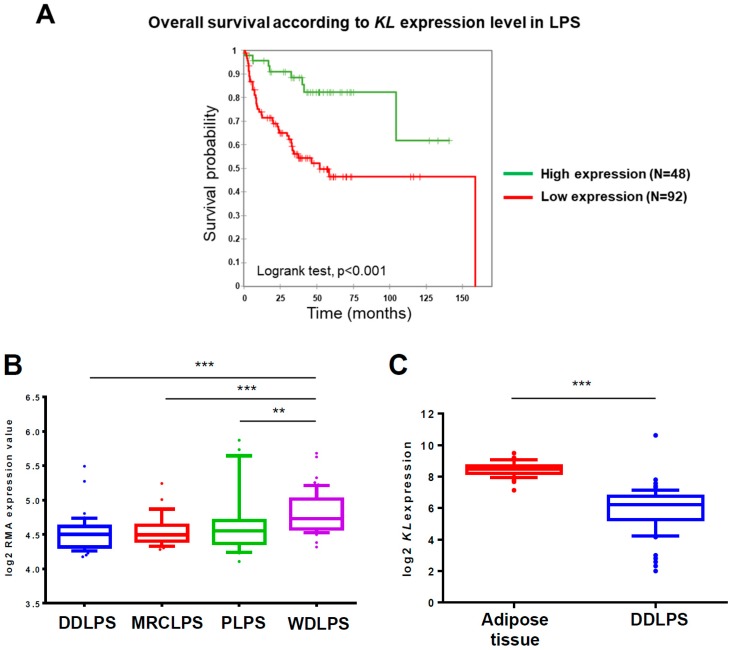
Klotho (*KL*) expression has a prognostic value for liposarcoma patients and is downregulated in dedifferentiated liposarcomas (DDLPS) tumors (**A**) Kaplan-Meier curves of the overall survival in 140 liposarcoma patients divided into high- and low-risk groups according to the expression level of *KL* in tumors (high or low, cut-off = mean) determined by microarrays (dataset ID: GSE30929). Higher *KL* expression is significantly associated with a better survival for liposarcomas (LPS) patients (Log-rank test, *p* < 0.001). (**B**) A boxplot of *KL* mRNA expression in dedifferentiated (DDLPS, *n* = 40), myxoid round cell (MRCLPS, *n* = 28), pleomorphic (PLPS, *n* = 20), and well-differentiated (WDLPS, *n* = 52) liposarcomas profiled on a gene expression microarray (dataset ID: GSE30929). *KL* expression is significantly (** *p* < 0.01 and *** *p* < 0.001) higher in WDLPS tumors compared to other histotypes. (**C**) A boxplot of *KL* mRNA expression in adipose tissue (*n* = 49) and DDLPS tumors (*n* = 61) profiled on gene expression microarray (GSE13506 and GSE21050, respectively) and normalized by GENT database. *KL* expression is significantly (*p* < 0.001) reduced in DDLPS tumors compared to adipose tissue. On the boxplots, the line in the middle of the box is the median; the whiskers are drawn down to the 10th percentile and up to the 90th. Points below and above the whiskers are represented as individual points.

First, we examined whether *KL* expression has a clinical relevance in terms of survival for LPS patients. A Kaplan Meier analysis of 140 primary human LPS samples profiled on gene expression microarrays (GSE30929) revealed a significant difference in survival times between the two groups dividing patients according to *KL* expression level in tumors (Figure 1A). In the same cohort, we compared histotypes and noticed that the *KL* expression is higher in WDLPS compared to other tumors (Figure 1B). We next focused on DDLPS because of their relatively high prevalence and chemoresistance.

Since *KL* is expressed in normal adipose tissue [26], we investigated whether its expression is significantly altered in DDLPS tumors. For that purpose, we used free publicly available data from the GENT database, which provides processing and normalization of datasets produced by different laboratories (Figure 1C). The data pointed out that *KL* is significantly downregulated in the 61 DDLPS samples (GSE21050) analyzed by a gene expression microarray as compared to the 49 samples of normal adipose tissue (GSE13506). As for now, unfortunately, there is no available dataset on *KL* expression in tumors at the protein level. These results altogether show that *KL* mRNA expression is downregulated in DDLPS, compared to normal tissue and well-differentiated tumors, and that a lower expression is associated with a poorer prognosis for LPS patients. This turns Klotho into a potential clinical biomarker in high-grade LPS and suggests that its loss could be a selective advantage for tumor development. We hypothesized that this might be related to its action on IGF-1R signaling [27].

### 2.2. In the DDLPS Cell Line, Klotho Reduces IGF-1R-Dependent Signaling

**Figure 2:**
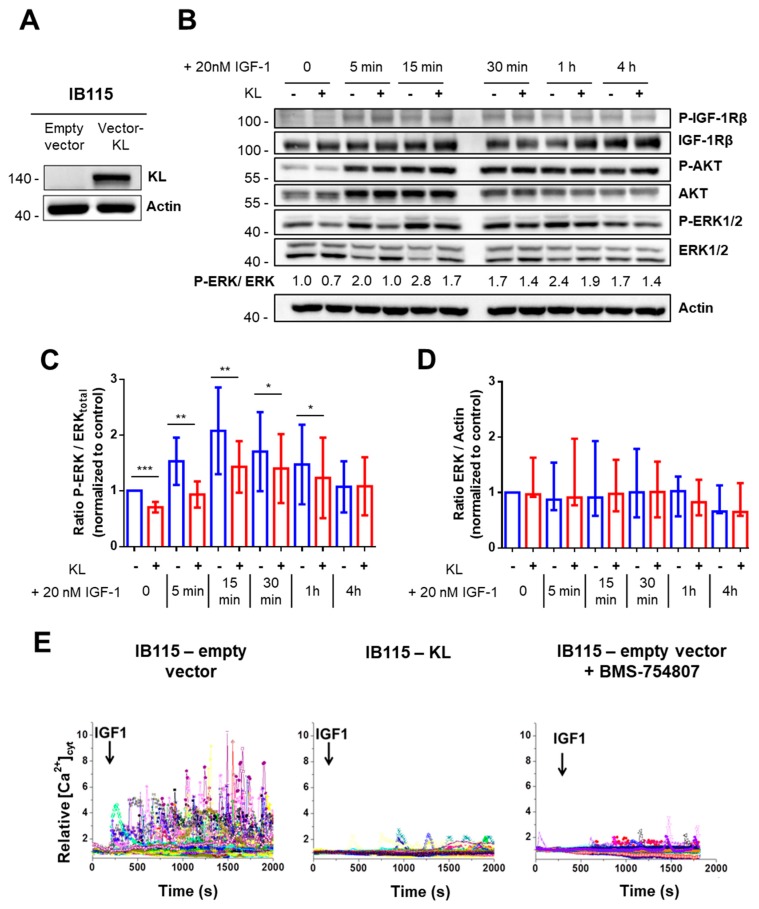
In the DDLPS cell line, Klotho reduces IGF-1R-dependent signaling (**A**) Validation of *KL*-overexpression in the IB115 cell line by western blotting after lentiviral transduction. The 135 kDa-band was not detected in the control cell line expressing the empty vector. Actin was used as a loading control. (**B**) Western blot analysis of IGF-1Rβ, AKT, and ERK1/2 phosphorylation statuses during stimulation with 20 nM IGF-1. For the comparison of the ratios between the phosphorylated form and total protein during treatment, the results were all normalized to the control condition (IB115-empty vector, *t*_0_) set to 1. Actin was used as the loading control. Images shown are representative of at least three independent experiments. (**C**) Evolution of the ratio between phosphorylated ERK1/2 and the total ERK1/2 (normalized to control condition set to 1) during IGF-1 (20 nM) stimulation. Histograms sum up six independent experiments and correspond to the mean ± Standard Deviation (SD) (* *p* < 0.05, ** *p* < 0.01 and *** *p* < 0.001). (**D**) Evolution of the total ERK1/2 abundance (normalized to control condition set to 1) during IGF-1 (20 nM) stimulation. Data shown summarize six independent experiments and correspond to medians with the interquartile range (IQR). (**E**) Variations of relative cytosolic Ca^2+^ concentration ([Ca^2+^]_cyt_) were monitored by fluorescence video imaging in Fluo2-loaded cells bathed in HBSS (2 mM Ca^2+^) medium, pretreated or not during 24 h with BMS-754807 (50 nM). Human IGF-1 (20 nM) was added in the bath medium at 200 s. Each trace corresponds to the evolution of [Ca^2+^]_cyt_ in one cell. Data shown are representative of three independent experiments.

To assess the cellular role of Klotho in high-grade LPS, we established a DDLPS cell line stably expressing *KL* (IB115-KL) (Figure 2A). No *KL* expression was detected by western blotting in the control cell line (IB115-empty vector). Then, we investigated whether Klotho inhibits IGF-1 signaling in these cells.

Ligand binding induces IGF-1R auto-phosphorylation and activates two major pathways: the Ras-Raf-mitogen activated protein kinase (MEK)-ERK (MAPKs) and the phosphatidylinositol 3-kinase (PI3K)/AKT pathways. In addition, it also promotes an intracellular Ca^2+^-response whose effectors remain to be clearly elucidated but include PI3K [28,29] and MEK activity (Supplemental Figure S1A).

First, AKT and ERK1/2 phosphorylation during IGF-1 stimulation were analyzed by western blotting (Figure 2B). In contrast to previously published work on breast cancer [27], no difference regarding IGF-1R auto-phosphorylation on Tyr1131 and no reproducible AKT inhibition were observed between the control and *KL*-overexpressing cells. However, in resting conditions, Klotho significantly reduced the ERK1/2 phosphorylation ratio about 30% compared to the control cell line (Figure 2B,C). Moreover, upon IGF-1 stimulation, the ERK1/2 phosphorylation ratio remained significantly higher in IB115-empty vector cells than in IB115-KL cells until 1 h of treatment. Maximal ERK1/2 phosphorylation was reached 15 min after IGF-1 addition, with a ratio of around 2.1 and 1.4 for control and *KL*-overexpressing cell lines, respectively. Of note, ERK1/2 abundance did not significantly vary across time and between cell lines (Figure 2D). Therefore, in the IB115 cell line, Klotho reduces ERK1/2 phosphorylation level and prevents its hyperactivation during IGF-1 stimulation.

Then, we studied the cytosolic Ca^2+^-response to IGF-1 (Figure 2E). Whereas control cells responded by large Ca^2+^ oscillations, only rare and small Ca^2+^ transients were obtained in *KL*-overexpressing cells. Pretreating control cells with BMS-754807, a specific inhibitor of IGF-1R/insulin receptor (Supplemental Figure S1B), mimicked the response profile of IB115-KL cells (Figure 2E). Klotho has been described as an inhibitor of Transient Receptor Potential Canonical 6 (TRPC6) channel by blocking its exocytosis stimulated by serum growth factors as IGF-1 (through PI3K) and reducing its plasma membrane expression [30,31,32]. Therefore, we investigated the implication of TRPC6 in an IGF-1-induced Ca^2+^-response (Supplemental Figure S1C). No cytosolic oscillations were observed in IB115 cells stimulated with IGF-1 in a Ca^2+^-free medium, thus showing that the Ca^2+^-entry at the plasma membrane is necessary for an IGF-1-induced Ca^2+^-response. Furthermore, pretreating control cell line with larixyl acetate, the specific inhibitor of TRPC6, prevented cytosolic Ca^2+^ oscillations following IGF-1 stimulation.

This demonstrates that Klotho abrogates an IGF-1-induced Ca^2+^-response, which requires TRPC6-dependent currents and is not completely inhibited by MEK inhibition (Supplemental Figure S1A). So, in addition to ERK1/2, Klotho might eventually inhibit PI3K signaling independently of AKT activation.

### 2.3. KL Overexpression in a DDLPS Cell Line Decreases Its Tumorigenic Phenotypes

Since *KL* overexpression affects IGF-1R signaling in DDLPS cells, we investigated its effect on several tumoral phenotypes modulated by IGF-1R including cell proliferation, clonogenicity, and chemoresistance.

**Figure 3:**
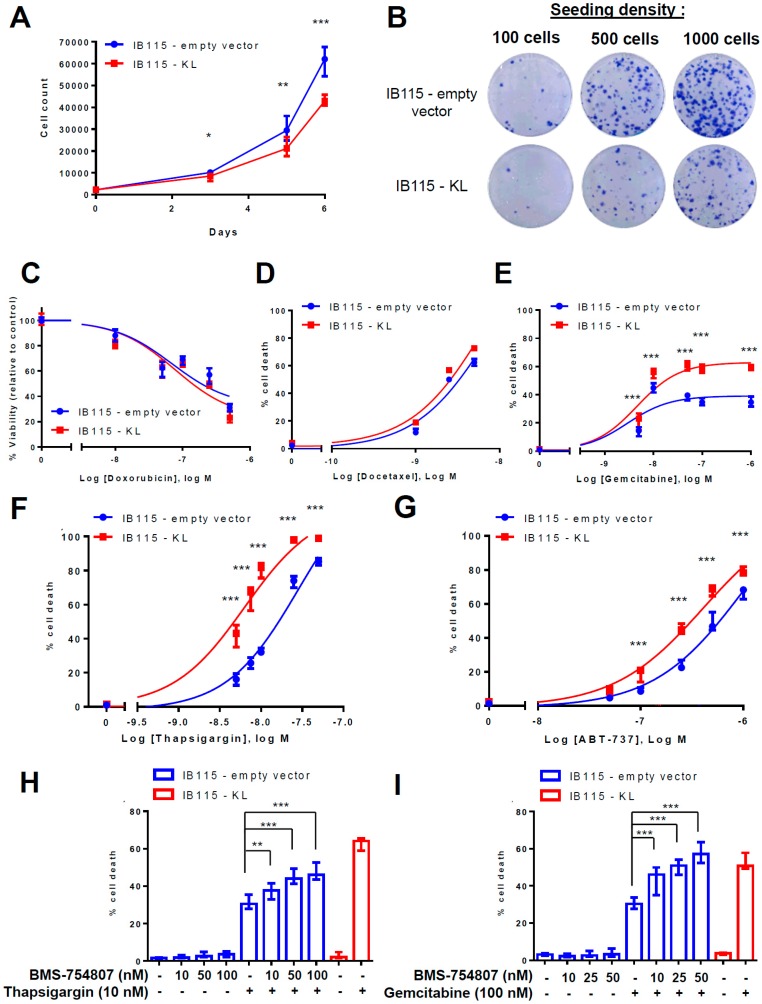
*KL* overexpression in a DDLPS cell line decreases its tumorigenic phenotypes. (**A**) Cell proliferation was assessed by flow cytometry as described in Material and Methods. *KL* significantly decreased the cell number at days 3, 5, and 6 (with * *p* < 0.05, ** *p* < 0.01 and *** *p* < 0.001, respectively). Results summarize the three independent experiments and correspond to the medians with IQR. (**B**) Clonogenicity was measured by crystal violet staining in accordance with the protocol explained in the Material and Methods. Data shown are representative of the results obtained in three independent experiments. (**C**) The viability of IB115 cell lines after 72 h of incubation with several concentrations of doxorubicin was assessed by MTT assay. The results are represented as a percentage of the control condition (with the median and IQR) and summarize three independent experiments. (**D**–**G**) Mitochondrial potential loss assay was evaluated by flow cytometry with TMRM-loaded cells after 72 h of incubation with (**D**) docetaxel, (**E**) gemcitabine, (**F**) thapsigargin (TG), and (**G**) ABT-737, according to the protocol described in the Material and Methods. Compared to corresponding conditions with IB115-empty vector cells, *KL*-overexpression significantly (*p* < 0.001) enhanced cell death induced by gemcitabine, thapsigargin, and ABT-737. Dose-response curves were interpolated as standard slopes. The data shown summarize the results obtained in at least three independent experiments and correspond to median and IQR. Cell death of IB115 cell lines was measured by TMRM-staining and flow cytometry. IB115 cell lines were pretreated for 1 h with indicated concentrations of BMS-7548074 and then treated for 72 h with (**H**) 10 nM thapsigargin (TG) or (**I**) 100 nM gemcitabine. Compared to non-pretreated IB115-empty vector cells, BMS-7548074 significantly increased the TG and gemcitabine-induced cell death in a dose-dependent manner. Histograms sum up (**H**) four and (**I**) three independent experiments. Data shown correspond to medians with IQR.

*KL* overexpression significantly decreased cell proliferation from the third day on, resulting in about 30% reduction of cell count (on average 61,204 and 42,993 cells for the IB115-empty vector and IB115-KL cell lines, respectively) after 6 days of cell culture (Figure 3A). Moreover, IB115-KL cells had fewer clonogenic capacities than the control cell line (Figure 3B), thus confirming its tumor suppressor role. On the other hand, the main cytotoxic molecules used for LPS patients were screened (Figure 3C–E). After 72 h treatment, cell death was assessed using TMRM-staining (gemcitabine, docetaxel) or MTT assay (doxorubicin). No relevant difference between the two cell lines was observed with doxorubicin (Figure 3C) and docetaxel (Figure 3D). In contrast, *KL* overexpression significantly sensitized to gemcitabine-induced cell death (Figure 3E). *KL* overexpression does not decrease EC50 but rather enhances the magnitude of the plateau observed on the dose-response curve: maximal cell death rates reach about 39% and 63% in the IB115-empty vector and IB115-KL cells, respectively.

Gemcitabine targets dividing cells and differently alters cell cycle depending on the concentration, as described by Cappella and collaborators [33] and noticed in IB115 cells (Supplemental Figure S2). Indeed, after its activation by phosphorylation, gemcitabine has a biphasic effect: first a cytostatic and then a cytotoxic effect. This explains the delayed apoptosis, but alterations of cell cycle vary from one concentration to another. Considering that Klotho does not interfere with the cell cycle disruption induced by gemcitabine (Supplemental Figure S2), we hypothesized that, after 72 h of treatment, its pro-apoptotic action mainly occurs at moderate (100 nM) and high concentrations (100 µM) of gemcitabine at the end of the cytostatic phase and during the recovery phase. The fact that Klotho promotes caspase-dependent apoptotic signaling was confirmed with a dual AnnexinV/PI staining (Supplemental Figure S3).

These results are consistent with previous work showing that IGF-1 signaling protects cells from gemcitabine-induced cell death in pancreatic cancer [34]. It is worth noting that IGF-1 signaling is also well-known for protecting cells from ER stress-induced apoptosis by increasing the adaptive capacity of ER [35]. ER stress was already implicated in resistance to gemcitabine [36,37]. Therefore, we tested the hypothesis that, by inhibiting IGF-1 signaling, Klotho could sensitize DDLPS cells to ER stress-inducing agents. Therefore, DDLPS cell lines were incubated with thapsigargin (TG), an inhibitor of Sarco-Endoplasmic Reticulum Ca^+^-ATPase (SERCA) pumps (Figure 3F) and thus, an ER stress inducer. TMRM-staining demonstrates that Klotho dramatically sensitizes DDLPS cells to TG-induced cell death (EC50 = 28.2 vs. 6.32 nM for IB115-empty vector and IB115-KL cells, respectively). Similar results were obtained with ABT-737, a BH3-mimetic that inhibits anti-apoptotic proteins and consequently increases the Ca^2+^ transfer from ER to mitochondria, thus reducing reticular Ca^2+^ content [38] (Figure 3G). Indeed, the EC50 of ABT-737 was shifted from 995 nM in IB115-empty vector cells to 407 nM in IB115-KL cells.

These results indicate the specific role of Klotho in gemcitabine and ER-stress-induced apoptosis in DDLPS cells. In order to confirm IGF-1R implication in resistance to apoptosis, the control cell line was incubated with sub-lethal concentrations of BMS-754807 (Figure 3H,I). IGF-1R inhibition significantly increased the cell death caused by TG (Figure 3H) or gemcitabine (Figure 3I) in a dose-dependent manner.

These data confirm that IGF-1R signaling, which is partially inhibited by Klotho, promotes DDLPS cell survival during TG and gemcitabine treatments.

### 2.4. KL-Overexpression Sensitizes DDLPS to ER Stress by Disrupting Intracellular Calcium Homeostasis

**Figure 4:**
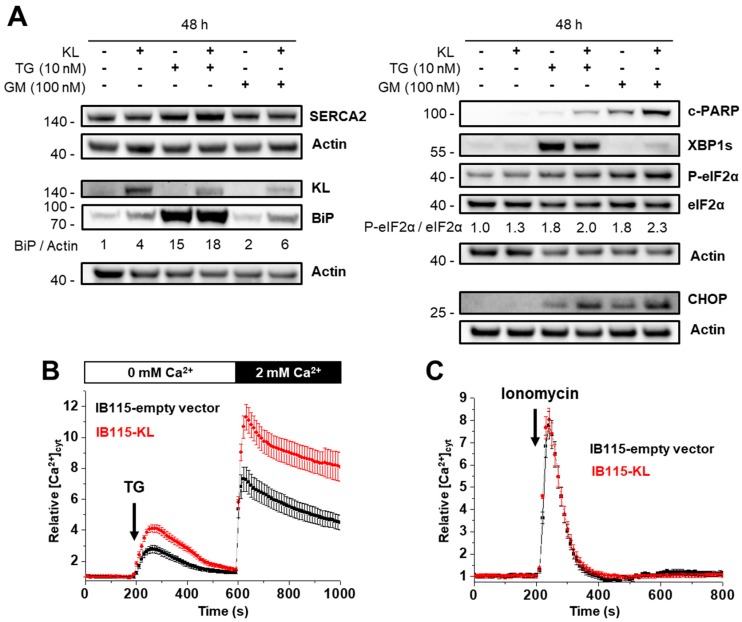

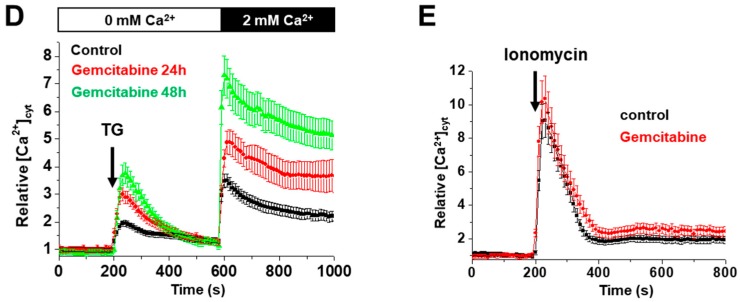
KL-overexpression sensitizes DDLPS to endoplasmic reticulum (ER) stress by disrupting the intracellular Ca^2+^ homeostasis. (**A**) Abundance and phosphorylation ratios of proteins implicated in ER stress were analyzed by western blotting after 48 h of incubation with no drugs, 10 nM thapsigargin (TG) or 100 nM gemcitabine. Actin was used as a loading control. To compare the ratios between phosphorylated form and the total protein during treatments, the results were all normalized to control condition (IB115-empty vector, no treatment) set to 1. Results are representative of three independent experiments. (**B**–**E**) Variations of relative cytosolic Ca^2+^ concentration were monitored by fluorescence videomicroscopy in Fluo2-loaded cells bathed in a Ca^2+^-free HBSS medium. Results are represented as means ± SD. (**B**,**D**) TG (10 nM) was added at 200 s to evaluate the intracellular Ca^2+^ mobilization and (**B**) 2 mM Ca^2+^ was applied at 600 s to measure the capacitive Ca^2+^-entry. (**C**,**E**) Ionomycin (100 nM) was added at 200 s to visualize the ER Ca^2+^-content. (**B**) *KL* overexpression increased intracellular Ca^2+^ response to TG and the subsequent capacitive Ca^2+^ entry (*n* = 143 and *n* = 183 for the IB115-empty vector and IB115-cells, respectively), but (**C**) did not affect the Ca^2+^ ER content, compared to IB115-empty vector cell line (*n* = 79 and *n* = 123 for the IB115-empty vector and IB115-cells, respectively). (**D**,**E**) IB115-empty vector cells were pretreated with gemcitabine (100 nM) during 24 and 48 h. Gemcitabine increased (**D**) TG-induced Ca^2+^ response (control *n* = 96; 24 h, *n* = 82; 48 h, *n* = 34) but (**E**) did not alter the size of the reticular Ca^2+^ pools (control *n* = 102; gemcitabine *n* = 81).

Klotho might sensitize cells to TG by affecting the SERCA expression. According to gene expression microarray analysis, SERCA1 and SERCA3 genes are not (or only a few) transcribed while the SERCA2 gene is highly expressed in IB115 cells (Supplemental Figure S4). Furthermore, in adipose tissue, no SERCA1 protein is detected and SERCA3 is only slightly expressed according to the Human Protein Atlas database. In contrast, SERCA2 is upregulated in LPS compared to normal tissues and high SERCA2 expression is associated with high malignant LPS such as DDLPS [39,40]. Through western blotting, we found that Klotho does not affect the expression of SERCA2 (Figure 4A).

Long-term exposition to TG induces ER stress and activation of the Unfolded Protein Response (UPR), which encompasses several pathways that activate both cell death and survival mechanisms. The balance between these pathways determines cell fate and is modulated by IGF-1 signaling. In pancreatic cancer, actors of the UPR are activated by gemcitabine and some of them, especially XBP-1 (X-box binding protein 1), are implicated in chemoresistance [36,37]. We hypothesized that Klotho might sensitize DDLPS cells to apoptosis by affecting ER stress pathways.

The analysis of the activation of several arms of the UPR (Figure 4A) confirmed that gemcitabine treatment activates ER stress signaling in the IB115 cell line, although it has only a modest effect on the expression of the chaperone protein GRP78/BiP (Binding immunoglobulin Protein), which is a well-known marker of ER stress and so, is massively expressed during TG treatment.

Interestingly, Klotho enhances BiP expression (Figure 4A), even in resting conditions with about a 4-fold increase, suggesting either a basal reticular stress in these cells or an effect related to the Ca^2+^-binding activity of BiP in the lumen of the ER.

During the TG treatment, *KL* overexpression reduces XBP-1 splicing but enhances the eIF2α activation as illustrated by the stronger induction of the pro-apoptotic CHOP (Figure 4A), an observation that is consistent with increased apoptosis and reduced IGF-1 signaling [41]. With gemcitabine, this effect of Klotho on eIF2α-dependent signaling is also observed, while the detection of spliced XBP-1 here rather reflects a more intense ER stress. An increased cell death in *KL*-overexpressing cells is illustrated by higher PARP cleavage (Figure 4A).

Enhanced ER stress in IB115-KL cells might originate from a depletion of reticular Ca^2+^. To address whether Klotho increases the ER stress by modulating the ER Ca^2+^-content, we measured the relative cytosolic Ca^2+^ concentration ([Ca^2+^]_cyt_) in cells bathed in a Ca^2+^-free medium and applied TG (Figure 4B), which passively depletes ER stores, or ionomycin (Figure 4C), a Ca^2+^ ionophore that reveals the size of the Ca^2+^ stores. Strikingly, the Ca^2+^-release following TG treatment was greatly increased in *KL*-overexpressing cells, as the subsequent capacitive Ca^2+^-entry (Figure 4B), whereas the Ca^2+^-response to ionomycin was similar in both cell lines. These data suggested that the *KL* overexpression enhances reticular Ca^2+^-leak but has no effect on the size of the Ca^2+^ stores. Direct assessment of reticular calcium concentration ([Ca^2+^]_ER_) with a genetic Ca^2+^-sensor specifically targeted to ER confirmed that Klotho neither alters resting [Ca^2+^]_ER_ nor enhances the reticular Ca^2+^ pools releasable by ionomycin, but significantly enhances the amplitude of the [Ca^2+^]_ER_ decrease induced by TG (Supplemental Figure S5).

This implies that, in resting conditions, an increased Ca^2+^-leakage is compensated by active ER re-filling, probably through the increased activity of SERCA pumps. On the other hand, Ca^2+^ released into cytosol might probably be buffered by mitochondria. The estimation of mitochondrial Ca^2+^-content by addition of CCCP (mitochondrial oxidative phosphorylation uncoupler) confirmed a greater accumulation of Ca^2+^ in mitochondria of IB115-KL cells (Supplemental Figure S6A). Moreover, the application of TG into the extracellular medium induced a significantly higher increase of relative mitochondrial [Ca^2+^] in *KL*-overexpressing cells compared to the control cell line (Supplemental Figure S6B). Therefore, during the long-term TG treatment, increased cell death observed in IB115-KL cells may originate from increased ER stress (through reticular Ca^2+^-stores depletion) and a probable mitochondrial Ca^2+^ overload.

Afterward, we investigated the effect of gemcitabine on reticular Ca^2+^-content of control cell line after 24 and 48 h of treatment (Figure 4D,E). Intracellular Ca^2+^-mobilization following TG addition and subsequent capacitive Ca^2+^-entry are increased in a time-dependent manner (Figure 4D): the amplitude of Ca^2+^-responses have doubled after 48 h of treatment with gemcitabine. However, no significant difference is observed with ionomycin (Figure 4E). So, gemcitabine-induced ER stress is associated with a reticular Ca^2+^-leak that increases over time.

In this context, increased capacitive Ca^2+^-entry seems to be a consequence of enhanced intracellular Ca^2+^-mobilization rather than a direct target of Klotho. Moreover, the inhibition of Calcium Release-Activated Channels (CRAC) by BTP2 did not protect IB115-KL cells from gemcitabine-induced cell death (Supplemental Figure S7). Actually, BTP2 even tended to sensitize control cell line to gemcitabine, although the differences were not statistically significant. So, the enhanced capacitive Ca^2+^-entry might be mainly involved in intracellular stores refilling and does not play a major role in gemcitabine-induced cell death.

To conclude, TG application revealed that Klotho promotes a constitutive reticular Ca^2+^-leakage. This probably contributes to the greater sensitivity to ER stressors such as TG, ABT-737, or gemcitabine, resulting in a higher CHOP induction, possible mitochondrial Ca^2+^-overload, and cell death.

### 2.5. Intracellular Localization of TRPC6 Contributes to Reticular Ca^2+^-Leakage in KL-Overexpressing DDLPS Cells

In order to identify the molecular mechanisms by which Klotho increases reticular Ca^2+^-leakage, we first hypothesized that this could involve the Transient Receptor Potential Canonical 6 (TRPC6) channel. Indeed, in neural cancer cells, TRPC6 can be translocated to the reticulum and this localization results in an increase in passive Ca^2+^-efflux without any modification of the reticular Ca^2+^ content [42]. So, as previously reported in other models, Klotho could modulate the TRPC6 intracellular localization in DDLPS cells, thereby explaining its inhibitory action on the IGF-1-induced Ca^2+^-response.

**Figure 5:**
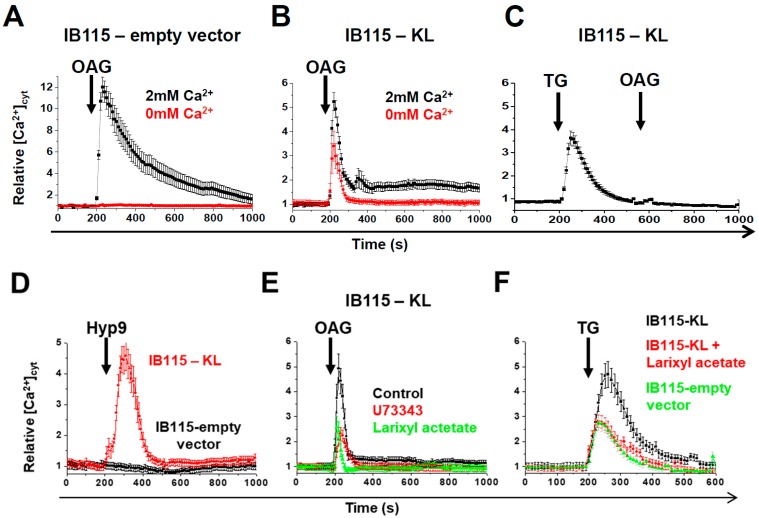

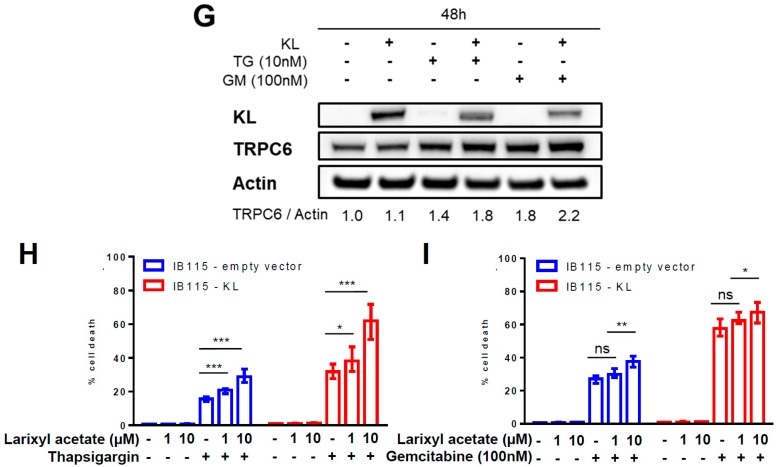
The intracellular localization of TRPC6 contributes to reticular Ca^2+^-leakage in *KL*-overexpressing DDLPS cells, but is not necessary to increase cell death. (**A**–**F**) Variations of relative cytosolic Ca^2+^ concentration were monitored by fluorescence videomicroscopy in Fluo2-loaded cells. Each experiment was repeated three times and the average of more than 20 single-cell traces was analyzed. The effect of OAG (50 µM) addition was evaluated in (**A**) IB115-empty vector or (**B**) IB115-KL cells bathed in HBSS medium ± 2 mM Ca^2+^, and in (**C**) IB115-KL cells pretreated with TG (10 nM) at 200 s in a Ca^2+^-free HBSS medium. (**D**) IB115 empty-vector and IB115-KL cells were bathed in a Ca^2+^-free HBSS medium and stimulated at 200 s by the specific activator of TRPC6 Hyp9 (1 μM). (**E**) OAG (50 µM) was added at 200 s to Ca^2+^-free HBSS medium on IB115-KL cells control or pretreated with inhibitors of TRPC6 (U73343 and larixyl acetate both at 1 μM, 1 h). (**F**) Ca^2+^-leakage was estimated by the application of 10 nM TG at 200 s on IB115-empty vector or IB115-KL cells, which were pretreated or not with larixyl acetate (1 μM, 1 h), in a Ca^2+^-free HBSS medium. (**G**) The abundance of TRPC6 was analyzed in IB115-empty vector and IB115-KL cells by western blotting after 48 h of incubation with no drugs, 10 nM TG, or 100 nM gemcitabine. Actin was used as a loading control. To compare TRPC6 abundance between conditions, results were all normalized to control conditions (IB115-empty vector, no treatment). Results shown are representative of three independent experiments. (**H**,**I**) Cell death was measured with a TMRM-staining analyzed by flow cytometry after 72 h incubation of IB115 cell lines, which were pretreated during 1 h with indicated concentrations of larixyl acetate and then treated with (**H**) 20 nM and 10 nM TG for the IB115-empty vector and IB115-KL cells, respectively (in order to have nearly similar cell death rates), or (**I**) 100 nM gemcitabine. Histograms sum up (**H**) four and (**I**) two independent experiments. Data shown correspond to medians (IQR).

To test this hypothesis, DDLPS cells were stimulated with 50 µM of OAG, a structural analog of DAG (DiAcylGlycerol) that activates Ca^2+^ entry through TRPC-3,-6 channels [43]. After the OAG addition, a large cytosolic [Ca^2+^] increase was observed in IB115-empty vector cells bathed in the Ca^2+^-containing medium, but not in Ca^2+^-free medium, meaning that the previous cytosolic Ca^2+^ increase was due to a Ca^2+^ influx through TRPC channels at the plasma membrane (Figure 5A). In IB115-KL cells bathed in a Ca^2+^-containing extracellular medium, OAG induced a smaller (about 50% decrease) transient Ca^2+^ peak, which was only partially affected by the absence of Ca^2+^ in the extracellular medium (Figure 5B). This suggests that in these cells, OAG mainly mobilized calcium ions stored in an intracellular compartment. The pretreatment of IB115-KL cells with TG abolished OAG-induced intracellular Ca^2+^ release (Figure 5C), suggesting that the OAG-sensitive channels are mainly located at the ER membrane and mobilize Ca^2+^ from TG-sensitive reticular stores.

To confirm the TRPC6 implication, the cells were bathed in a Ca^2+^-free medium and stimulated with Hyp9, a specific activator of TRPC6. Similar to OAG stimulation, a cytosolic Ca^2+^-response was recorded in IB115-KL but not the B115-empty vector cells (Figure 5D). The pretreatment of IB115-KL cells with TRPC6 inhibitors (U73343 or larixyl acetate) significantly reduced the amplitude of the Ca^2+^-response to OAG (Figure 5E) and to TG (Figure 5F), thus confirming the role of TRPC6 in both OAG-induced response and the passive reticular Ca^2+^-leak. Pretreatment with a specific inhibitor of TRPC3 (Pyr3) combined to larixyl acetate suppressed OAG-response (Supplemental Figure S8). Therefore, it can be hypothesized that the residual Ca^2+^-response to OAG in larixyl acetate-treated cells is mediated by TRPC3 channels. However, to our knowledge, there is no evidence of the role of TRPC3 at the ER or for a link between IGF-1, KL, and TRPC3 in the literature. So, the mechanisms involving TRPC3 would require further investigation. Comparing Ca^2+^-response of Pyr3- and larixyl acetate-treated cells suggests that TRPC3 is less implicated than TRPC6 in Ca^2+^-response to OAG (Supplemental Figure S8).

The analysis of TRPC6 abundance by western blotting revealed that TRPC6 expression is increased after 48 h incubation with 10 nM TG or 100 nM gemcitabine and, more importantly, in IB115-KL cells (Figure 5G). This suggests that TRPC6 might play a role in TG- and gemcitabine-induced cell death.

Though larixyl acetate (the specific TRPC6 inhibitor) reduced the TG-induced Ca^2+^-response, no protection against TG-induced apoptosis was observed in IB115-KL cells (Figure 5H). Actually, TRPC6 inhibition aggravated TG-induced apoptosis in both cell lines (Figure 5H), while it did not meaningfully modify cell death after gemcitabine treatment (Figure 5I). However, larixyl acetate does not allow for the distinction between respective roles of reticular- and plasma membrane-located TRPC6 channels. Previous studies reported that TRPC6 takes part in the Ca^2+^-entry induced by TG [44,45,46]. The sensitization to apoptosis observed in IB115-empty vector cells (Figure 5H) suggests that the influx of Ca^2+^ through the TRPC6 channels at the plasma membrane is involved in cell survival.

Despite the fact that intracellular TRPC6 channels take part in TG-induced Ca^2+^-release and so, probably promote ER stress, these last results altogether suggest that TRPC6 sequestration at the ER is probably not the only and major mechanism by which Klotho sensitizes to TG- and gemcitabine-induced cell death.

### 2.6. In DDLPS Cells, Klotho Promotes Reticular Ca^2+^ Leakage and Apoptosis by Opening the Translocon

According to the literature, the major effector of the reticular Ca^2+^-leak is the translocon (TLC) [47], a protein complex that allows for the translocation of nascent polypeptides into the ER lumen for elongation and maturation. When the polypeptidic chain is released, Ca^2+^ leaks through the pore of the TLC, which is, at that time, in an open state for Ca^2+^. Puromycin and anisomycin are antibiotics that inhibit protein synthesis but, respectively, open and close the TLC [47].

**Figure 6:**
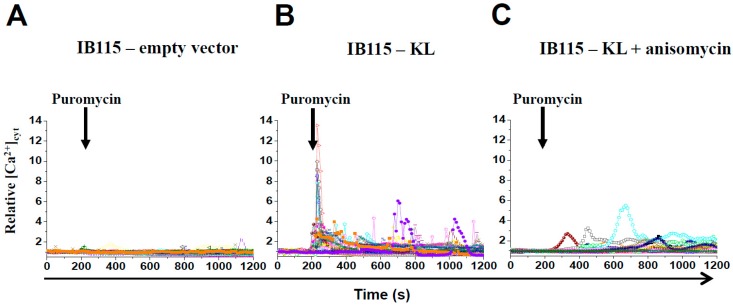

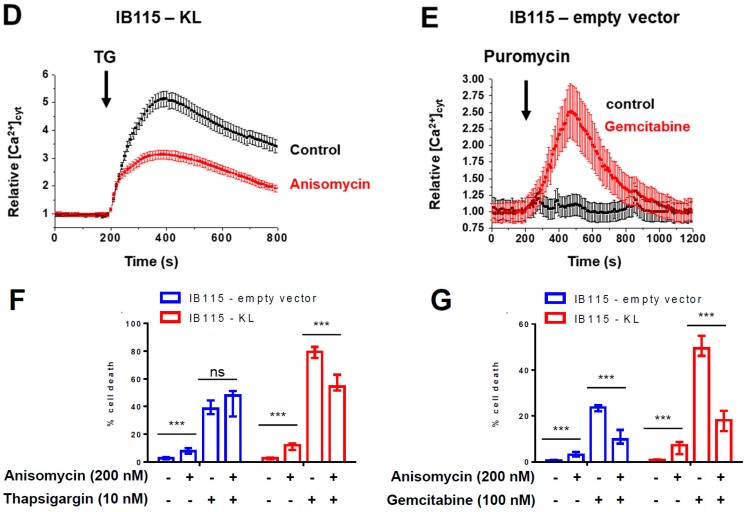
In DDLPS cells, Klotho promotes reticular Ca^2+^-leakage and apoptosis by opening the translocon. (**A**–**E**) Variations of relative cytosolic Ca^2+^ concentration were monitored by fluorescence videomicroscopy in Fluo2-loaded cells. Data shown are representative of three independent experiments. (**A**–**C**) In a Ca^2+^-free HBSS medium, puromycin (25 µg/mL) was applied at 200 s on (**A**) IB115-empty vector cells, (**B**) IB115-KL cells, and on (**C**) IB115-KL cells pretreated 30 min with 200 nM anisomycin, a translocon-closing molecule. Each trace corresponds to the evolution of [Ca^2+^]_cyt_ in one cell. (**D**) In a Ca^2+^-free HBSS medium, IB115-KL cells, pretreated (*n* = 114) or not (*n* = 76) with 200 nM anisomycin during 30 min, were stimulated at 200 s with TG (10 nM) to evaluate reticular Ca^2+^-leakage. Results are represented as means ± SD. (**E**) In a Ca^2+^-free HBSS medium, IB115-empty vector cells, pretreated (*n* = 78) or not (*n* = 81) with 100 nM gemcitabine during 24 h, were stimulated at 200 s with puromycin (25 µg/mL) to assess Ca^2+^-leakage through the translocon. Results are represented as means ± SD. (**F**,**G**) Cell death was measured by TMRM-staining and flow cytometry in IB115 cell lines pretreated for 1 h with 200 nM anisomycin and then incubated for 72 h with (**F**) 10 nM TG or (**G**) 100 nM gemcitabine. Histograms sum up (**F**) three and (**G**) four independent experiments. Data shown correspond to medians and IQR.

Interestingly, puromycin had no effect in IB115-empty vector cells (Figure 6A), whereas it elicited a huge transient increase in [Ca^2+^]_cyt_ followed by Ca^2+^-oscillations in IB115-KL cells (Figure 6B). These were drastically reduced by anisomycin pretreatment (Figure 6C). TLC closure by anisomycin significantly reduced the TG-induced Ca^2+^-release (Figure 6D), thereby confirming that TLC is implicated in the basal reticular Ca^2+^-leakage.

In addition, puromycin triggered a cytosolic Ca^2+^-response in IB115-empty vector cells pretreated with gemcitabine (Figure 6E). This demonstrates that the gemcitabine-induced Ca^2+^-leakage is mediated at least in part by opening the TLC.

Anisomycin significantly reduced the death rates of IB115-KL cells after 72 h incubation with TG (Figure 6F) or gemcitabine (Figure 6G). The protective effect towards gemcitabine in both cell lines was not due to protein synthesis inhibition because puromycin did the opposite and was counteracted by anisomycin on the wild-type IB115 cell line (Supplemental Figure S9A). Moreover, the long-term treatment with anisomycin did not affect *KL*-overexpression (Supplemental Figure S9B). Furthermore, anisomycin reduced BiP expression in resting conditions and during gemcitabine treatment, but not the other reticular chaperone protein calreticulin (Supplemental Figure S9B). To conclude, anisomycin reduces ER stress by limiting the TLC-mediated Ca^2+^-leakage, which is induced by gemcitabine and enhanced by Klotho.

Altogether, these results demonstrate that in *KL*-overexpressing cells, a higher proportion of TLCs is in an open-state. This contributes to the basal reticular Ca^2+^-leakage revealed by TG treatment and aggravates the gemcitabine-induced TLC opening. This results in reticular Ca^2+^-depletion in IB115-KL cells after 48 h of treatment (Supplemental Figure S9C), in contrast to control cells (Figure 4D,E), and in increased apoptosis.

All major results concerning the effect of Klotho on TLC opening and reticular stress were confirmed in two other DDLPS cell lines (IB111 and IB143) (Supplemental Figure S10). Indeed, *KL* overexpression increases BiP expression (Supplemental Figure S10A), Ca^2+^-response to puromycin (Supplemental Figure S10B) and to TG (Supplemental Figure S10C), and also cell death rates following long-term treatment with TG (Supplemental Figure S10D) or gemcitabine (Supplemental Figure S10E). To conclude, despite different genetic backgrounds and an intrinsic greater resistance to ER stress in IB111 and IB143 compared to IB115, Klotho increases TLC-opening and reticular Ca^2+^-leakage in both cell lines and significantly sensitizes them to cell death induced by TG or gemcitabine. Lower expression of KL in transduced IB111 and IB143 cells compared to IB115-KL cell line is one possible explanation for the more discrete effects on the phenotypes (Supplemental Figure S10A).

Then, we investigated which IGF-1 signaling pathway is implicated in the resistance to ER stressors and is inhibited by Klotho and addressed its impact on reticular Ca^2+^-leakage.

### 2.7. Klotho Regulates Drug Sensitivity and Reticular Ca^2+^-Leakage by Inhibiting ERK1/2 Signaling

**Figure 7:**
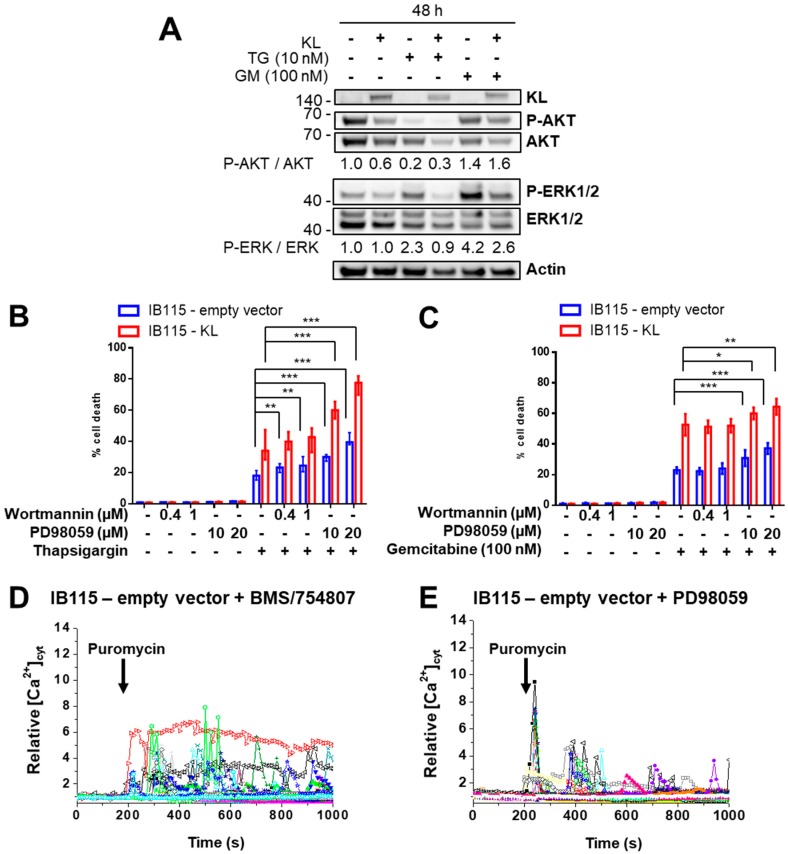

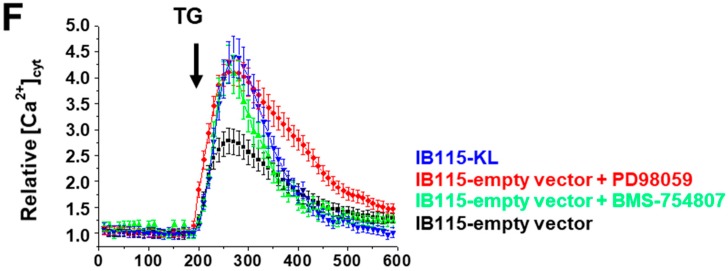
Klotho regulates drug sensitivity and reticular Ca^2+^-leakage by inhibiting ERK1/2 signaling (**A**) Analysis by Western blotting of the abundance of phosphorylated AKT and ERK1/2 compared to total protein expression, in IB115-empty vector and IB115-KL cells after 48 h of incubation with no drugs, 10 nM thapsigargin (TG) or 100 nM gemcitabine. Actin was used as a loading control. To compare the ratios between phosphorylated form and total protein during treatments, results were all normalized to the control condition (IB115-empty vector, no treatment) set to 1. Results are representative of three independent experiments. (**B**,**C**) Cell death was measured by TMRM-staining and flow cytometry. IB115 cell lines were pretreated for 2 h with Wortmannin or PD98059 as indicated and then incubated for 72 h with (**B**) 20 nM or 10 nM TG for IB115-empty vector and IB115-KL cells, respectively (in order to have an almost similar cell death), or (**C**) 100 nM gemcitabine. Histograms sum up (**B**) four (with median and IQR) and (**C**) three (represented as means ± SD) independent experiments. (**D**–**F**) Variations of relative cytosolic Ca^2+^ concentration were monitored by fluorescence videomicroscopy, in Fluo2-loaded cells bathed in a Ca^2+^-free HBSS medium. (**D**,**E**) Puromycin (25 µg/mL) was added at 200 s to IB115-empty vector cells pretreated with (**D**) BMS-754807 (50 nM, 24 h) or (**E**) PD98059 (1 µM, 2 h). Each trace corresponds to the evolution of [Ca^2+^]_cyt_ in one cell. Data shown are representative of three independent experiments. (**F**) TG (10 nM) was added at 200 s to IB115-KL cells (*n* = 83) and IB115-empty vector cells, which were not pretreated (*n* = 77) or pretreated with BMS-754807 (50 nM, 24 h, *n* = 78) or PD98059 (1 µM, 2 h, *n* = 75). Results correspond to means ± SD.

Two pathways of the IGF-1R signaling, ras/raf/MEK/ERK and PI3K/AKT, are known to play a role in the resistance to gemcitabine [48,49] and ER stress [50,51,52]. Since Klotho inhibits the IGF-1-induced Ca^2+^-response (mainly dependent on PI3K, see Supplemental Figure S1A) and ERK1/2 activation, we have studied the phosphorylation status of AKT and ERK1/2 after the TG and gemcitabine treatments (Figure 7A). AKT was de-phosphorylated after incubation with TG and only slightly phosphorylated after gemcitabine treatment. In contrast, ERK1/2 is phosphorylated in control cells after each treatment, but this activation is inhibited in *KL*-overexpressing cells. Thus, suggesting that ERK1/2 is implicated in resistance to TG and gemcitabine but not AKT.

This was confirmed with the use of PI3K (Wortmannin) and MEK (PD98059) inhibitors (Supplemental Figure S11). The Wortmannin pretreatment had only a slight effect on TG-induced cell death of IB115-empty vector cells (Figure 7B), but not of IB115-KL cells, and had no significant effect on apoptosis induced by gemcitabine (Figure 7C) in both cell lines. By contrast, PD98059 significantly potentiated the apoptotic response to TG and gemcitabine (Figure 7B,C) in both cell lines. These results altogether demonstrate that ERK1/2 but not AKT exerts a relevant pro-survival activity against ER stressors and is inhibited by Klotho in this DDLPS cell line.

Finally, we addressed the consequence of IGF-1R and MEK inhibition on reticular Ca^2+^-leakage. First, the pretreatment of control cells with BMS-754807 (Figure 7D) or PD98059 (Figure 7E) restored the reticular Ca^2+^-leakage induced by puromycin and observed in IB115-KL cells. Then, both inhibitors mimicked the effect of Klotho on TG-induced Ca^2+^-response (Figure 7F) without affecting the ER Ca^2+^-content (Supplemental Figure S12). Therefore, IGF-1R and, more precisely, the ERK inhibition is at least one mechanism by which Klotho promotes reticular Ca^2+^-leakage through the TLC and so, sensitizes these DDLPS cells to ER stressors releasing Ca^2+^ from reticular stores (such as TG, gemcitabine, and ABT-737).

## 3. Discussion

In this work, we evaluated the relevance of Klotho as a biomarker and as a tool for overcoming drug resistance in high-grade LPS.

We reported that a higher *KL* expression is associated with better survival of LPS patients. Moreover, its expression is detected at higher levels in WDLPS, which has a good prognosis. *KL* expression is critical for adipose tissue homeostasis but is altered in DDLPS, probably by epigenetic regulations as in several other cancers [53,54]. If in the future the normal tissue of each patient were to be systematically sampled during tumor excision, it would be interesting to confirm these clinical results at the protein level by comparing KL abundance in DDLPS tumors with matched-adipose tissue. Meanwhile, considering our observations at the transcriptomic level, *KL* downregulation may represent a selective advantage for tumor cells and so, may be associated with tumor evolution as for DDLPS.

In compliance with this observation, we did not detect, by western blotting, endogenous KL expression in any of the six DDLPS cell lines, isolated from the patients’ tumors, available in the lab. Moreover, for most of these cell lines, *KL* overexpression was rapidly eliminated by the transduced cells despite effective lentiviral transduction and cell selection.

To assess the cellular role of Klotho in DDLPS, we successfully established a DDLPS cell line stably overexpressing *KL*. Its inhibitory action on IGF-1-dependent Ca^2+^-signaling was shown through TRPC6 sequestration at the ER and on ERK1/2 activation. Analysis of tumor phenotypes indicated that Klotho reduces cell proliferation, clonogenicity, and has a major effect on drug sensitivity and Ca^2+^ homeostasis.

Because Ca^2+^ signaling is involved in several hallmarks of cancer, Ca^2+^ remodeling in cancer cells is a research field of growing interest [55]. Here, we have demonstrated for the first time, in three DDLPS cell lines, that Klotho increases reticular Ca^2+^-leakage by opening the TLC. This effect on the TLC is supported by the compensatory upregulation of BiP in *KL*-overexpressing cells because BiP interacts with the TLC complex and by this way limits Ca^2+^-leakage through the pore [56]. However, to the best of our knowledge, the link between the permeability of the TLC to Ca^2+^ and IGF-1R or ERK1/2 activity is unknown. This new and unexpected mechanism will require further investigation.

The constitutive Ca^2+^-leakage confers to the DDLPS cells overexpressing *KL* a greater susceptibility to apoptosis mediated by ER-stressors such as thapsigargin (TG), ABT-737, and gemcitabine, whose effect on TLC openings had never been described until now. This sensitization by Klotho is also related to a greater induction of CHOP, which promotes the transcription of pro-apoptotic genes and aggravates the reticular Ca^2+^-leakage [57]. In addition, an increased Ca^2+^-transfer from the ER to mitochondria might stimulate ATP production in resting conditions and promote ER refilling by SERCA pumps, but probably also results in mitochondrial Ca^2+^-overload and mitochondrial membrane potential loss during the exposure to these ER stressors.

These results are in conflict with previous work showing that Klotho protects from ER stress [58]. However, *KL*-overexpressing DDLPS cells share interesting similarities with *dwarf* fibroblasts during TG treatment, like increased apoptosis, reduced XBP-1 splicing, and preserved activation of eIF2α [41]. These fibroblasts were isolated from *Snell dwarf* mice, a longevity model that bears a homozygous mutation in the gene encoding Pit-1, resulting in several deficiencies in growth hormones including IGF-1. Indeed, insulin/IGF-1 signaling inhibition is a mechanism shared by many animal longevity models including *KL*-overexpressing mice [19,59]. Moreover, IGF-1 signaling was reported as a cytoprotective pathway against canonical ER stress [35,60], but also against gemcitabine [34] and ABT-737 [61]. All these observations are consistent with our results showing a potentiated apoptotic response to ER stressors mediated by the inhibition of IGF-1-dependent signaling in *KL*-overexpressing DDLPS cells. Therefore, the role of Klotho on ER stress might depend on a cellular context. Discrepancies between results might also arise from the fact that Banerjee et al. overexpressed mouse KL [58], which is not identical to human KL and could differ in terms of interactions with human proteins.

The inhibition of IGF1-R and MEK mimicked the effect of Klotho on Ca^2+^ homeostasis and sensitized to TG- and gemcitabine-induced cell death. Actually, there are numerous crosstalks between MAPK signaling and ER stress [62]. The MEK-ERK pathway was shown to promote cell survival during ER stress. ER stress signaling can also directly activate ERK1/2. Indeed, IRE-1 activation can stimulate ERK1/2 through an unknown mechanism. Reduced XBP-1 splicing in *KL*-overexpressing cells, which suggests a reduced IRE-1 activation, can be involved in attenuated ERK1/2 activation and so, in increased cell death. In addition, BiP can be translocated to the plasma membrane and activate the signaling cascade Ras-Raf-MEK-ERK. So, given that ERK1/2 is less activated in *KL*-overexpressing cells despite enhanced ER stress, Klotho may probably act through additional pathways in parallel to IGF-1R in order to avoid the hyperactivation of MAPK signaling. Considering that Ca^2+^ and the reactive oxygen species can modulate ERK1/2 activity and that Klotho regulates both, this provides new possibilities for investigation. Moreover, Klotho is a regulator of pathways that are commonly altered in cancer and activate MAPK, such as Wnt and FGF [25]. Its use in the therapeutic management of DDLPS is thus probably not restricted to IGF-1R inhibition.

Regarding reticular TRPC6, its activity as a passive leaky channel had already been reported but, until now, the only regulator identified for that mechanism was the Stromal Interacting Molecule 1 (STIM1) [42]. Here, we observed that the IGF-1-induced Ca^2+^ response requires TRPC6 and that MEK inhibition promotes the sequestration of active TRPC6 channels at the ER. So, the effects of Klotho on IGF-1-induced Ca^2+^ signaling and reticular Ca^2+^-leak through TRPC6 could be related to the regulation of STIM-1 by ERK1/2 [63]. Nevertheless, additional experiments are needed to test this hypothesis and physiological consequences remain to be studied.

For the first time, we bring evidence of a role for Klotho in LPS. If our results were confirmed in vivo, combining Klotho’s mechanism of action and ER stressors might be an alternative for DDLPS patient care. Indeed, LPS frequently overexpress SERCA2 and calreticulin [39,64] and, consequently, are sensitive to ER stressors [39]. By enhancing Ca^2+^-leakage-mediated ER stress, Klotho could increase the calreticulin exposure at the surface of tumor cells and so, facilitate immunogenic cell death. Considering the emerging improvements in immunotherapy, this question will certainly be addressed in the future.

## 4. Materials and Methods

### 4.1. Chemicals

Doxorubicin (Adriamycin; Pfizer, New York, NY, USA) and gemcitabine (Gemzar; Eli Lilly and Company, Neuilly-sur-Seine, France) were obtained from the pharmacy of the Bergonié Institute (Bordeaux, France). Docetaxel (Taxotere; Sanofi-Aventis, Gentilly, France) was obtained from Sigma-Aldrich (St. Quentin Fallavier, France). ABT-737 was purchased from Selleckchem (Munich, Germany).

About the other chemicals—thapsigargin, puromycin, anisomycin, ionomycin, Hyp9, OAG, U73343, and Wortmannin—were from Sigma-Aldrich. BMS-754807 and PD98059 were purchased from Selleckchem. Larixyl acetate was obtained from Santa Cruz Biotechnology (Dallas, TX, USA). Pyr3 was from Calbiochem (Merck Millipore, Darmstadt, Germany). Human IGF1 was from Miltenyi Biotec (Paris, France). QVD-OPh was purchased from R&D Systems (Minneapolis, MN, USA).

### 4.2. Cell Lines

The dedifferentiated liposarcoma cell lines IB111, IB115, and IB143 were a generous gift from F. Chibon (INSERM U1218, Bergonié Cancer Institute, Bordeaux) and were established and authenticated as previously described [65]. DDLPS and HEK-293T cell lines were respectively cultured in RPMI-1640/GlutaMAX-I and DMEM/GlutaMAX-I media (Life Technologies Inc., brand of ThermoFisher Scientific, Waltham, MA, USA) supplemented with 10% Foetal Bovine Serum (FBS). Cells were grown at 37 °C in a humidified atmosphere containing 5% CO_2_. Cultures were periodically tested for mycoplasma contamination.

IB111, IB115, and IB143 cells were infected with a lentiviral vector encoding human transmembrane KL (EX-Z9677-Lv105, GeneCopoeia, Rockville, MD, USA). Control cell lines were established with lentiviral transduction of an empty vector (pReceiver-Lv105, GeneCopoeia, Rockville, MD, USA). VSV-G-pseudotyped lentiviral particles were produced by co-transfection of HEK293T cells with previous vectors and the compatible packaging plasmids psPAX2 and pVSVg. Cell lines were incubated overnight with lentiviral supernatants and 8 μg/mL of polybrene (Sigma-Aldrich, St. Quentin Fallavier, France). Stably transduced cells were selected with puromycin (1 µg/mL, Sigma). Then, puromycin was removed from the culture medium. *KL* overexpression was regularly verified over passages by Western blotting.

### 4.3. Western Blot

For IGF-1 stimulation, the cells were harvested overnight before IGF-1 addition into an FBS-free culture medium. For protein extraction, cells were rinsed with ice-cold PBS and lysed for 30 min at 4 °C in RIPA lysis and extraction buffer (Sigma) supplemented with a protease/phosphatase inhibitor cocktail (Sigma-Aldrich, St. Quentin Fallavier, France), 5 mM sodium orthovanadate (New England Biolabs, Ipswich, MA, USA), 1 mM EGTA, and 10 mM sodium fluoride (Sigma-Aldrich, St. Quentin Fallavier, France). Lysates were pelleted 10 min at 17,000× *g*, 4 °C and supernatants were collected for protein quantitation (DC protein assay kit, Biorad, Hercules, CA, USA). After denaturation, 25 µg total proteins of each sample were separated by sodium dodecyl sulfate–polyacrylamide gel electrophoresis and transferred to polyvinylidene difluoride membranes (iBlot2, ThermoFisher Scientific, Waltham, MA, USA) for immunoblotting with the appropriate primary antibody at 4 °C overnight. Anti-KL antibodies were purchased from Sigma-Aldrich (St. Quentin Fallavier, France) (#SAB2104815 and #SAB2105026) and ThermoFisher Scientific (Waltham, MA, USA) (PA5-21078). Antibodies for detection of P-IGF1Rβ (Tyr1131) (#3021), p-AKT (Ser473) (#4060), AKT (#4685), p-ERK1/2 (Thr202/Tyr204) (#9101), ERK1/2 (#9101), BiP (#3177), p-eIF2α (Ser51) (#3597), XBP-1s (#12782), c-PARP (Asp214) (#5625), SERCA2 (#4388) and calreticulin (#12238) were obtained from Cell Signaling Technology (Danvers, MA, USA). Antibodies for IGF-1Rβ (#sc-713), eIF2α (#sc-11386), and CHOP (#sc-793) were from Santa Cruz Biotechnology (Dallas, TX, USA). Anti-TRPC6 (#ACC-120) was provided by Alomone Labs (Jerusalem, Israel). Anti-actin (#A2066) was from Sigma-Aldrich (St. Quentin Fallavier, France). After washing, blots were incubated for 1 h with a horseradish peroxidase-linked anti-rabbit antibody (Sigma-Aldrich, St. Quentin Fallavier, France) and processed for a chemiluminescent substrate (ThermoFisher Scientific, Waltham, MA, USA) according to the manufacturer’s instructions. The signal was detected using Fusion Fx7 (ThermoFisher Scientific, Waltham, MA, USA) imaging system. Actin was used as a loading control and the quantification of protein abundance was performed by blot densitometry using the ImageJ 1.48v software (Rasband, W.S., ImageJ, U. S. National Institutes of Health, Bethesda, MD, USA).

### 4.4. Cell Proliferation

A total of 2500 cells were plated into 24-well plates in four replicates. Culture medium was changed at days 0-3-5 (0.5 mL per well). After trypsinization, the cell number was evaluated by flow cytometry (FACS Calibur, BD Biosciences, San Jose, CA, USA) based on their morphological features (FSC/SSC). Data acquisition and analysis were performed with the BD PlateManager and BD CellQuestPro software.

### 4.5. Clonogenic Assay

Totals of 100, 500, and 1000 cells were plated onto 6-well plates. Fresh culture medium was added every 2 to 3 days. After 10 days, cells were washed with PBS, fixed with 70% ethanol, washed again and then stained with a 1% Crystal Violet solution (Sigma-Aldrich, St. Quentin Fallavier, France). After several rinses with water and drying, the plates were scanned.

### 4.6. MTT Assay

A total of 5000 cells were seeded into 96-well plates in six replicates. The day after, the culture medium was replaced by a doxorubicin-containing medium (0.2 mL per well). After 72 h, 20 µL of 5 mg/mL MTT substrate (Sigma-Aldrich, St. Quentin Fallavier, France) solution were added to each well. Plates were incubated 2 h at 37 °C in a humidified atmosphere containing 5% CO_2_. Then, the medium was discarded and formazan was dissolved in DMSO. Absorbance of each well was measured with the microplate reader FlexStation 3 (Molecular Devices, San Jose, CA, USA) and calculated (A_570nm_–A_630nm_). For each condition, the viability was calculated as a percent of control. Dose-response curves were interpolated with Prism6 v6.01 (GraphPad Software Inc, La Jolla, CA, USA) software as standard slopes.

### 4.7. Mitochondrial Potential Loss Assay (Tetramethylrhodamine, Methyl Ester, TMRM)

A total of 5000 cells were seeded into 96-well plates in at least three replicates. The day after, the culture medium was replaced by drug-containing medium (0.2 mL per well). After 72 h of incubation, 200 nM TMRM dye (Fluka, brand of ThermoFisher Scientific, Waltham, MA, USA) and 20 µM verapamil (Sigma-Aldrich, St. Quentin Fallavier, France) were added into medium. Plates were incubated at 37 °C, 5% CO_2_ for 30 min. Then, supernatants were collected, cells were trypsinized and resuspended in their respective supernatants. TMRM-staining was analyzed by flow cytometry (FACS Calibur, BD Biosciences, San Jose, CA, USA). Data acquisition and analysis were performed with the BD PlateManager and BD CellQuestPro software. Cell population was determined on a FSC/SSC dot plot. Then, the region corresponding to cells that lost their mitochondrial electric potential (TMRM-negative cells) was selected on a SSC/FL2 dot plot diagram. The number of TMRM-negative cells is shown as a percentage of the analyzed cell population. Dose-response curves were interpolated with Prism6 v6.01 (GraphPad Software Inc., La Jolla, CA, USA) software as standard slopes.

### 4.8. Cytosolic Calcium Imaging

For calcium imaging, 100,000 cells were seeded onto glass coverslips (25 mm dia.) and incubated in a complete culture medium for 48 h. Single-cell cytosolic calcium imaging was performed, using Fluo2 LR-AM calcium dye as previously described [66]. Fluorescence intensity changes were normalized to the initial fluorescence value F_0_ and expressed as F/F_0_ (relative [Ca^2+^]_cyt_). One field was acquired from each coverslip and the data were pooled from six independent coverslips on three different days. Data were processed using OriginPro 7.5 software (Origin Lab, Northampton, MA, USA). On graphs, data were summarized as the mean ± standard deviation (SD).

### 4.9. Statistical Analysis

The cohort of 140 primary liposarcomas is from Gobble et al. [67] (GSE30929). Data about patient survival, histotypes and *KL* expression values (estimated with robust multi-array average (RMA)) were obtained with the web tool GEO2R. Patients were divided into groups according to the *KL* expression value (“high” or “low”, cut-off = mean). Survival rates were estimated using the Kaplan–Meier method and compared using the log-rank test using XL-STAT v2018.1 (Addinsoft, Paris, France) software.

Each experiment was repeated at least three times. To examine the statistical significance of the results, analyses were performed with the Prism6 v6.01 (GraphPad Software Inc, La Jolla, CA, USA) software. The normal distribution of datasets was examined with a Shapiro–Wilk normality test. If the data passed the normality test, the statistical significance between the two conditions was assessed with an unpaired *t*-test and the results were represented as the mean ± standard deviation (SD). Otherwise, a Mann–Whitney test was used and the median with interquartile range (IQR) was plotted. For western blot experiments, paired tests were used. Significant differences are represented as * if *p*-value *p* < 0.05, ** if *p* < 0.01 and *** if *p* < 0.001.

## 5. Conclusions

In this study, we evidence that Klotho is downregulated in DDLPS tumors and that its re-expression in DDLPS cell lines enhances the apoptotic response to ER stressors. Its mechanism of action mainly relies on the opening of the TLC, which mediates reticular Ca^2+^ leak and is induced by at least the attenuation of the IGF-1/ERK signaling pathway. Further experiments should be performed in order to identify the molecular players modulating TLC opening and regulated by ERK and Klotho. This would provide new therapeutic targets for overcoming drug resistance of DDLPS.

## Supplementary Materials

The following are available online at http://www.mdpi.com/2072-6694/10/11/439/s1, Figure S1: Pharmacological inhibition of IGF-1 signaling in IB115 cells, Figure S2: Klotho has no effect on cell cycle alterations induced by gemcitabine treatment, Figure S3: Impact of KL expression on caspase-dependent cell death induced by gemcitabine at 100 nM during 72 h, Figure S4: Distribution of ATP2A1-3 genes expressions represented by probe intensities, Figure S5: Reticular Ca^2+^ concentration [Ca^2+^]ER was assessed with the genetic biosensor ErGAP1, Figure S6: Klotho increases mitochondrial Ca^2+^ content and TG-induced mitochondrial Ca^2+^ uptake, Figure S7: Capacitive Ca^2+^ entry has no significant impact on gemcitabine-induced cell death, Figure S8: Impact of TRPC3,6 channels inhibition on the response to OAG in IB115-KL cells, Figure S9:Anisomycin reduces ER stress whereas gemcitabine depletes the reticular Ca^2+^ stores of IB115-KL cells, Figure S10: Sensitization to ER stressors by TLC opening was confirmed in two other DDLPS cell lines overexpressing *KL*, Figure S11. Western blot analysis of AKT and ERK1/2 phosphorylation status in IB115-empty vector cells after 24 h incubation with no drug, BMS-754807 (50 nM), Wortmannin (1 µM) or PD98059 (20 µM), Figure S12. Pretreatments with BMS-754807 or PD98059 does not affect reticular Ca^2+^ content.
