# Supplementary material for: The Role of the Anti-Aging Protein Klotho in IGF-1 Signaling and Reticular Calcium Leak: Impact on the Chemosensitivity of Dedifferentiated Liposarcomas
Source: Cancers (Basel). 2018 Nov 14;10(11):439. doi: 10.3390/cancers10110439 (PMC6266342; doi:10.3390/cancers10110439)
Supplement: Supplementary file 1 [file cancers-10-00439-s001.pdf]

Supplementary information: Role of the Anti-Ageing Protein Klotho in IGF-1 Signaling and in Reticular Calcium Leak: Impact on the Chemosensitivity of Dedifferentiated Liposarcomas

Vanessa Delcroix, Olivier Mauduit, Nolwenn Tessier, Anaïs Montillaud, Tom Lesluyes, Thomas Ducret, Frédéric Chibon, Fabien Van Coppenolle, Sylvie Ducreux and Pierre Vacher

Supplemental figures:

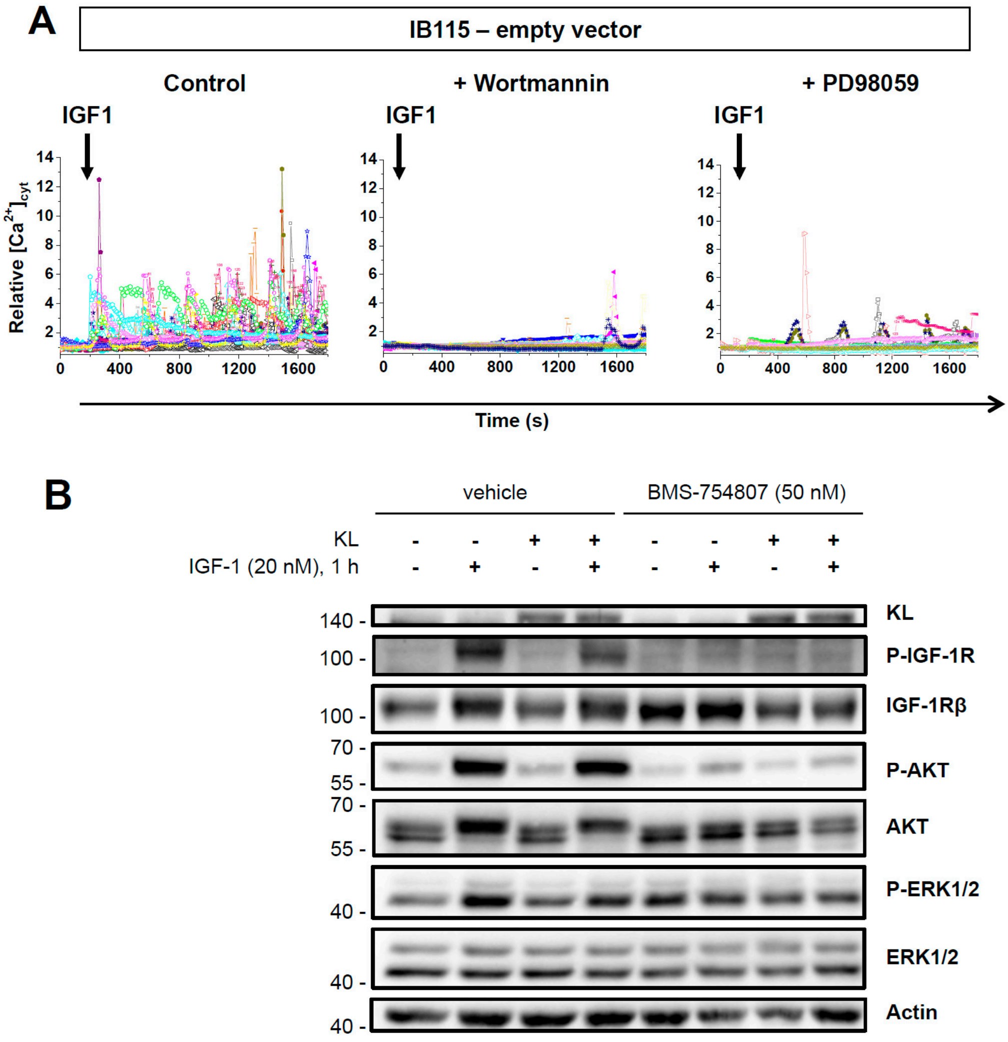

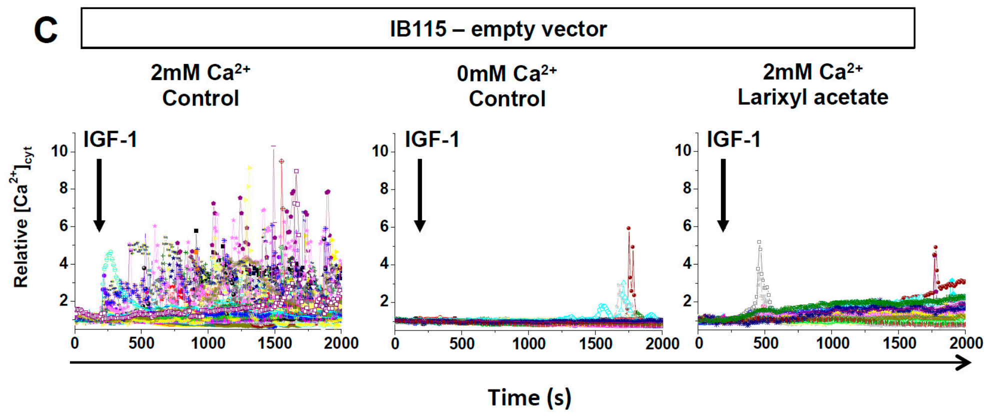

Figure S1: Pharmacological inhibition of IGF-1 signaling in IB115 cells (**A**) Variations of relative cytosolic Ca^{2+} concentration were monitored, by fluorescence video imaging in Fluo2-loaded IB115-empty vector cells bathed in HBSS (2 mM Ca^{2+}), without any pretreatment (control), or pretreated with Wortmannin (1 µM, 2 h) or PD98059 (10 µM, 2 h). IGF-1 (20 nM) was added in the bath medium at 200 s. Each trace corresponds to the evolution of $[Ca^{2+}]_{cyt}$ in one cell. Data shown are representative of three independent experiments. PD98059 reduced the number of responding cells and the magnitude of responses, whereas almost no cytosolic response was observed in Wortmannin-pretreated cells. (**B**) Western blot analysis of IGF-1Rβ, AKT and ERK1/2 phosphorylation status in IB115-empty vector and IB115 KL cells after 24 h incubation in FBS-free medium with vehicle (DMSO) or BMS-754807 (50 nM) and then 1 h stimulation with 20nM IGF-1. Actin was used as loading control. BMS-754807 pretreatment efficiently repressed phosphorylation of IGF-1R, AKT and ERK1/2 induced by IGF-1. (**C**) Variations of relative cytosolic Ca^{2+} concentration were monitored, by fluorescence video imaging in Fluo2-loaded IB115-empty vector cells bathed in HBSS (2 mM Ca^{2+}) without any pretreatment (control, n = 58), bathed in Ca^{2+}-free HBSS medium (n = 38) or bathed in HBSS (2 mM Ca^{2+}) and pretreated with larixyl acetate (1 µM, 1 h, n = 40). IGF-1 (20 nM) was added in the bath medium at 200 s. Each trace corresponds to the evolution of $[Ca^{2+}]_{cyt}$ in one cell. Data shown are representative of three independent experiments. Extracellular Ca^{2+} and TRPC6 activity are required for IGF-1-induced Ca^{2+}-response in DDLPS cells.

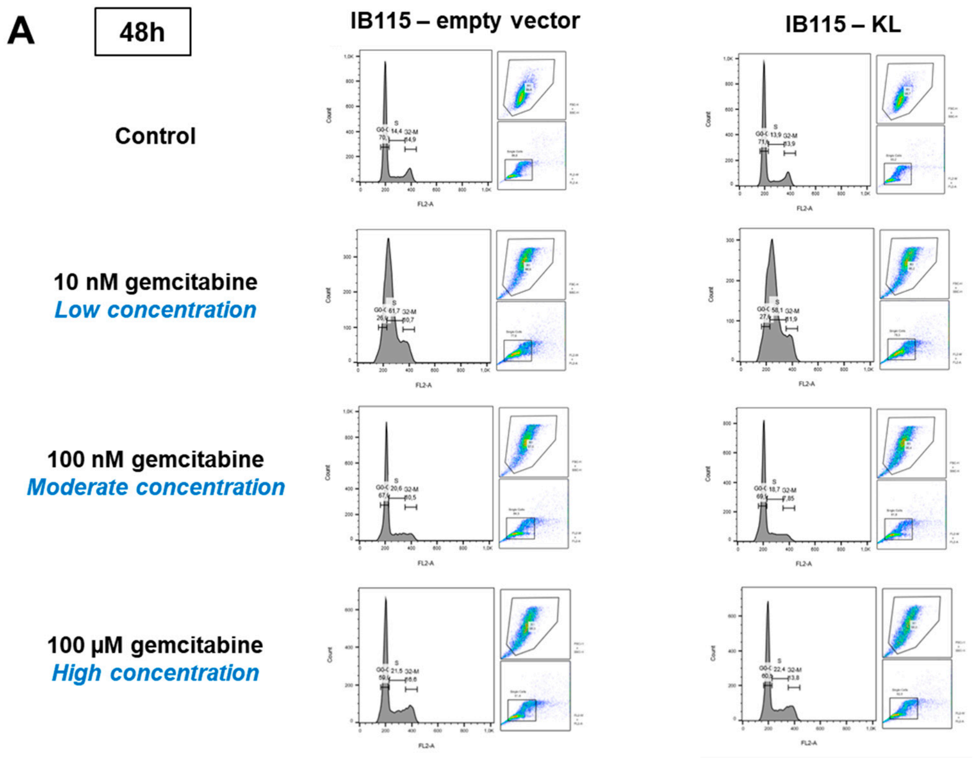

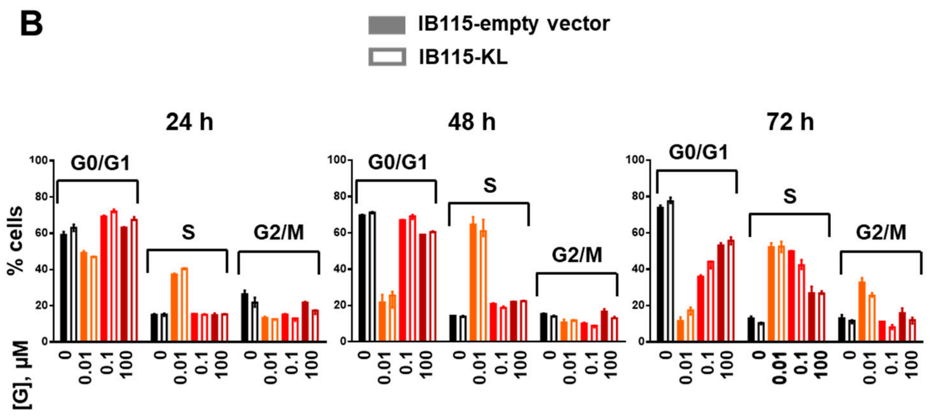

Figure S2: Klotho has no effect on cell cycle alterations induced by gemcitabine treatment. (**A**) Representative DNA content distributions obtained after incubation of cells during 48 h in culture medium, supplemented or not with gemcitabine at 10 nM, 100 nM or 100 µM. (**B**) Proportion of cells in each cell cycle phase depending on treatment duration and gemcitabine concentration [G]. Histograms summarize results that are representative of three independent experiments. Color-filled bars are for IB115-empty vector cells, white bars are for IB115-KL cells. Data are represented as median ± range.

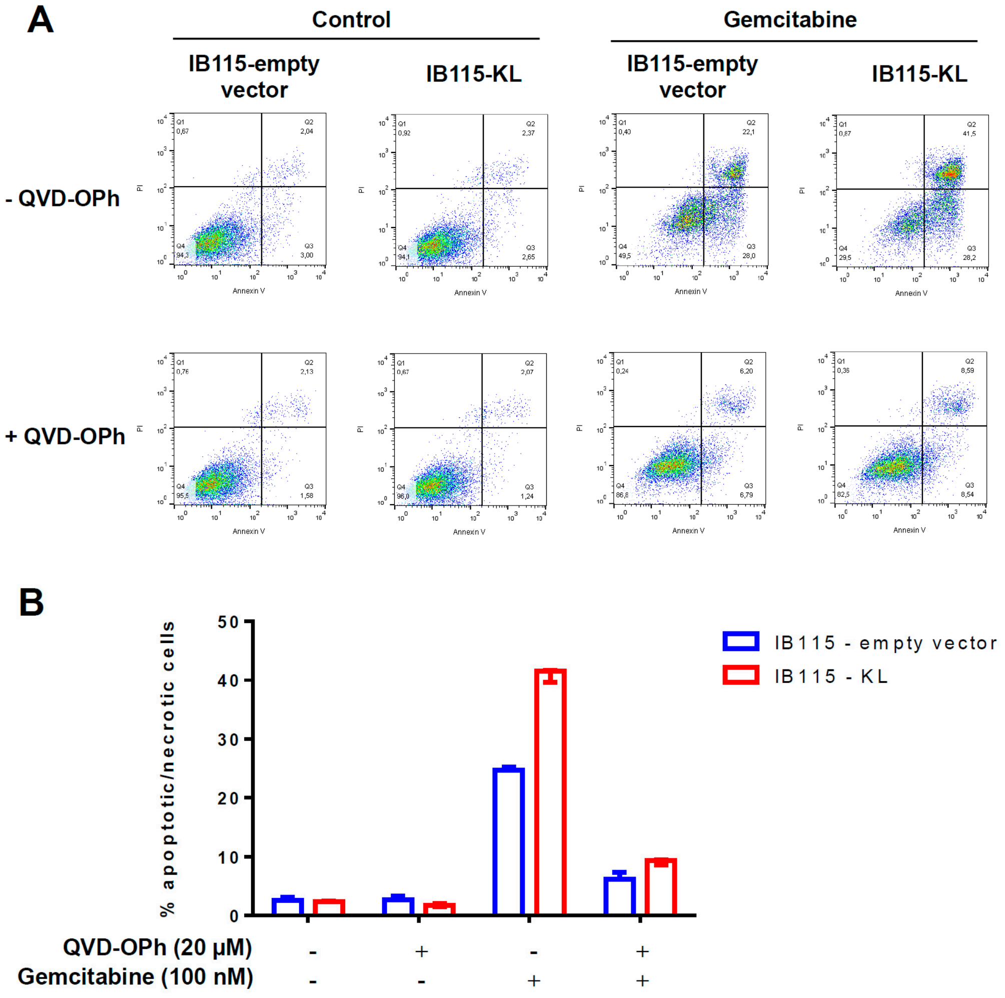

Figure S3: Impact of *KL* expression on caspase-dependent cell death induced by gemcitabine at 100 nM during 72 h. (**A**) Representative quadrants obtained for each condition. (**B**) Histograms summarize results that are representative of three independent experiments. Data are represented as median ± range.

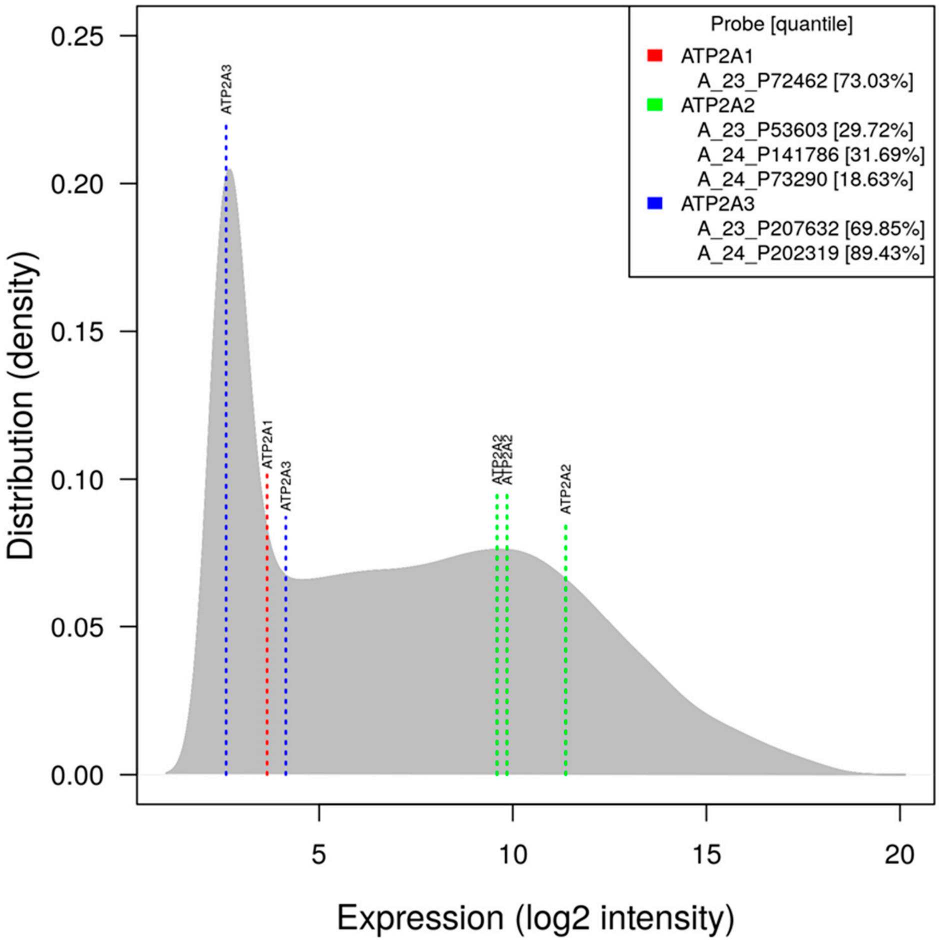

Figure S4: Distribution of ATP2A1-3 genes expressions represented by probe intensities. ATP2A1, ATP2A2 and ATP2A3 probes are highlighted with matching quantiles, which represent their expression levels compared to all probes in the array. This indicates a low expression for ATP2A1 and ATP2A3 genes (nearby the background expression) and a higher expression for ATP2A2.

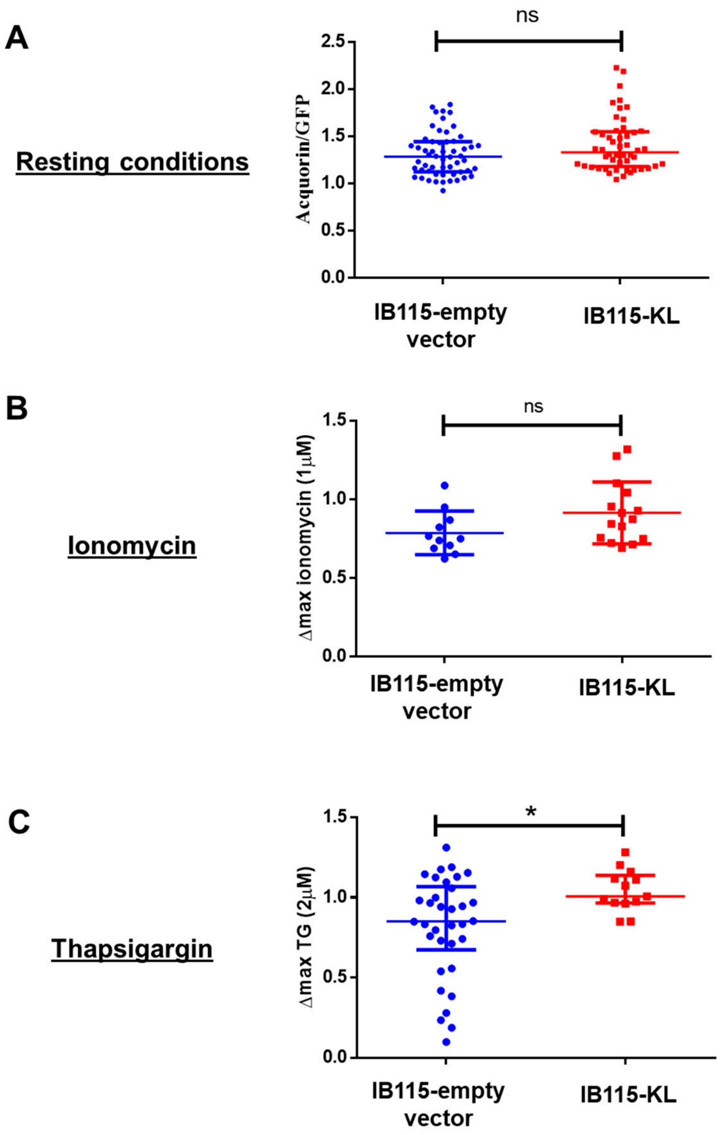

Figure S5: Reticular Ca^{2+} concentration $[Ca^{2+}]_{ER}$ was assessed with the genetic biosensor ErGAP1. (**A**) In resting conditions, reticular Ca^{2+} levels are similar in both cell lines. Amplitude of $[Ca^{2+}]_{ER}$ decrease was measured by subtracting minimal value to basal fluorescence intensity after addition of (**B**) ionomycin (1 μM) or (**C**) thapsigargin (2 μM). Reticular Ca^{2+} depletion is significantly ($p < 0.05$) higher in IB115-KL cells only after TG addition, thus confirming increased reticular Ca^{2+} leakage. Data shown sum up at least three independent experiments and correspond to median and IQR.

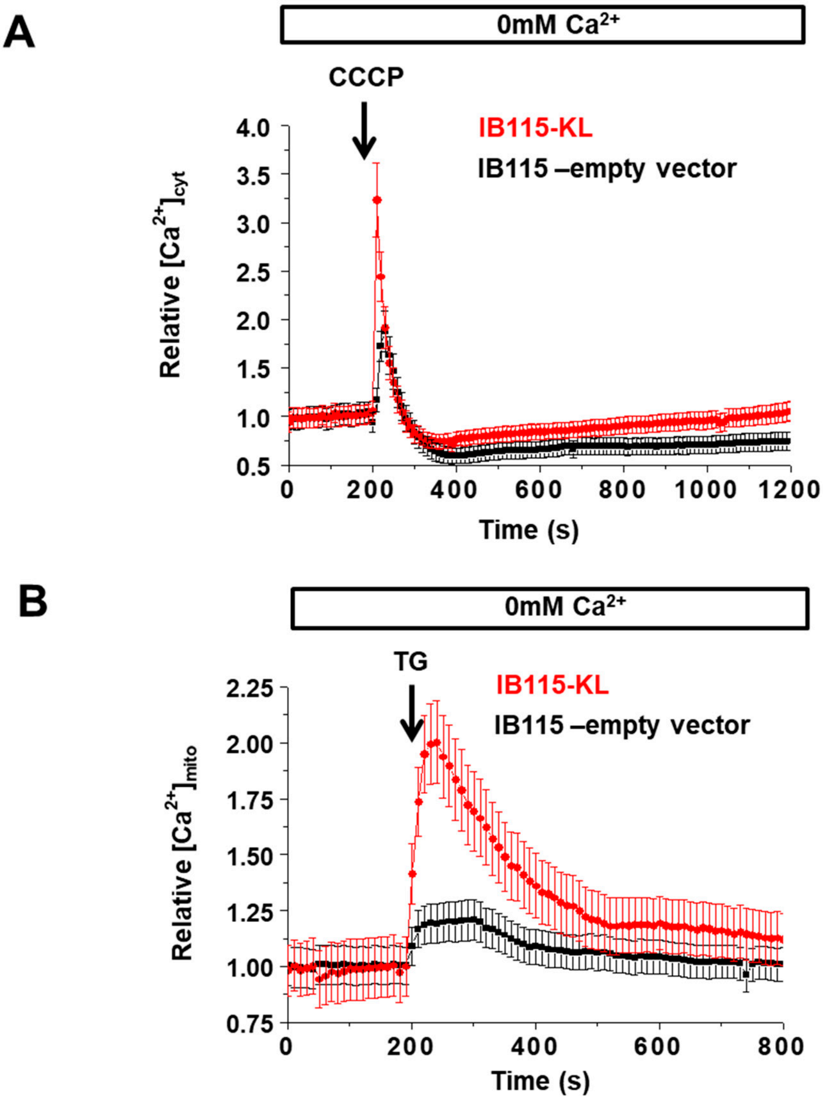

Figure S6: Klotho increases mitochondrial Ca^{2+} content and TG-induced mitochondrial Ca^{2+} uptake. Variations of relative Ca^{2+} concentration were monitored, by fluorescence video imaging in (**A**) Fluo2- and (**B**) Rhod2-loaded cells. Cells were bathed in Ca^{2+}-free HBSS. (**A**) CCCP (1 μM) was added in the bath medium at 200 s to evaluate mitochondrial Ca^{2+} content in IB115-empty vector ($n = 60$) and IB115-KL ($n = 63$) cells. (**B**) TG (10 nM) was added at 200 s to compare Ca^{2+} accumulation in mitochondria between control ($n = 72$) and *KL*-overexpressing ($n = 75$) cell lines. Data shown are representative of three independent experiments.

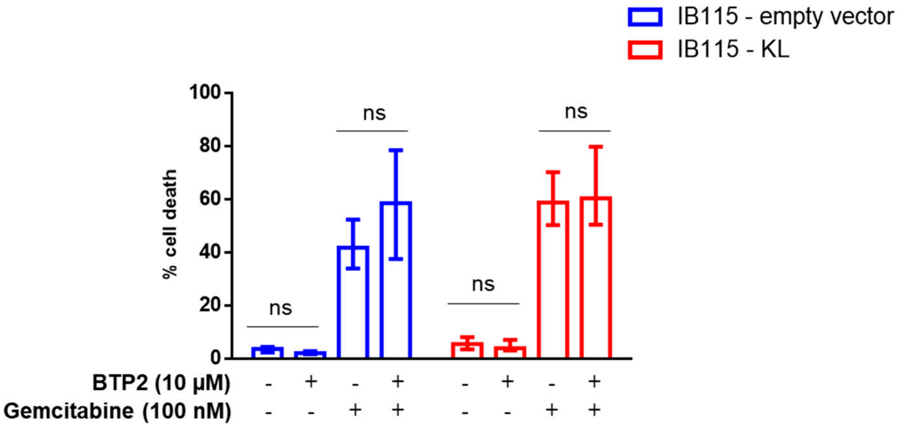

Figure S7: Capacitive Ca^{2+} entry has no significant impact on gemcitabine-induced cell death. To assess the role of capacitive Ca^{2+} entry in gemcitabine-induced cell death, cell lines were pretreated or not for 1 h with BTP2 (10 µM) and then incubated during 72 h with 100 nM gemcitabine. Cell death was measured by TMRM-staining, analyzed by flow cytometry. Data shown sum up two independent experiments with three replicates and correspond to median with range.

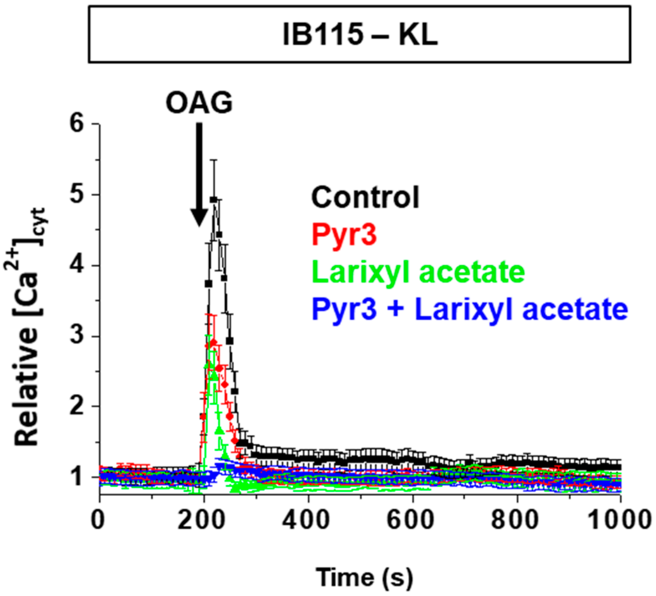

Figure S8: Impact of TRPC3,6 channels inhibition on the response to OAG in IB115-KL cells. Variations of relative cytosolic Ca^{2+} concentration were monitored, by fluorescence videomicroscopy in Fluo2-loaded cells. IB115-KL cells were bathed in Ca^{2+}-free HBSS, without any pretreatment (control, *n* = 122), pretreated with Pyr3 (1 µM, 1 h, *n* = 41), or larixyl acetate (1 µM, 1 h, *n* = 74) or with Pyr3 and larixyl acetate (both at 1 µM, 1 h, *n* = 42). OAG (50 µM) was added in the bath medium at 200 s. Data shown are representative of three independent experiments and correspond to mean ± SD. Pyr3 reduced cytosolic Ca^{2+} increase, but less potently than larixyl acetate. Combined inhibitors abrogated OAG-induced Ca^{2+}-response.

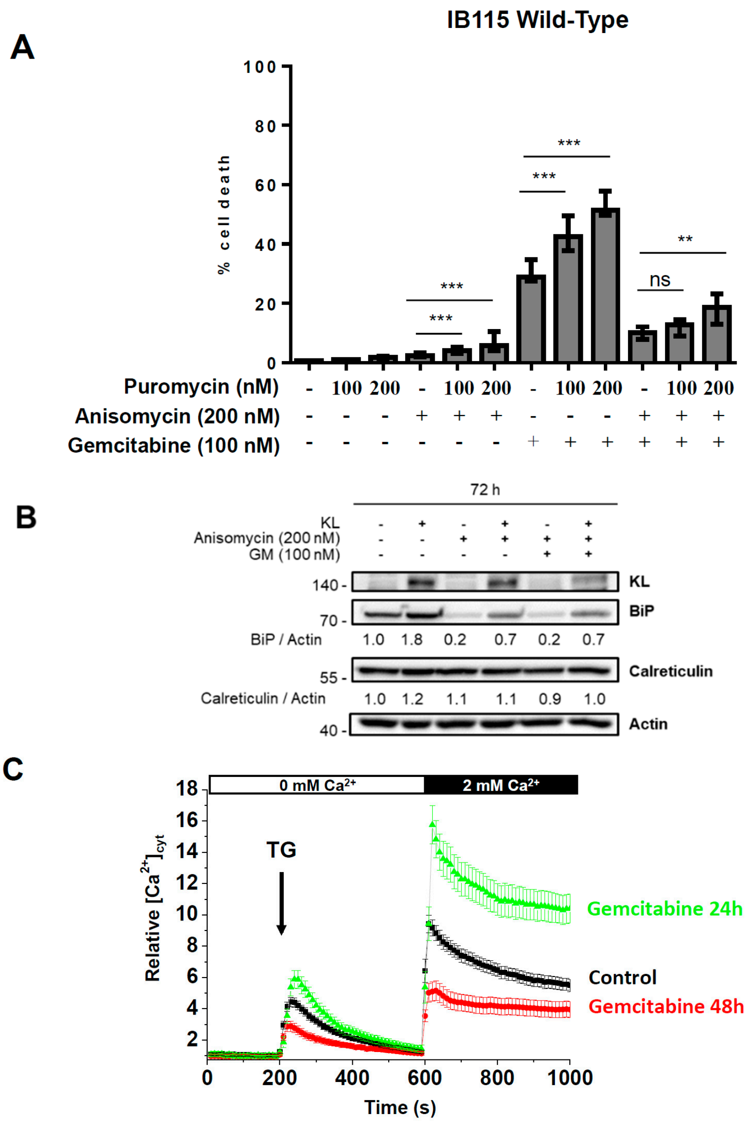

Figure S9: Anisomycin reduces ER stress whereas gemcitabine depletes the reticular Ca^{2+} stores of IB115-KL cells (**A**) Since the vector, expressed by transduced cells, contains the puromycin-resistance gene, IB115 parental cell line (wild-type) was pretreated or not for 1 h with anisomycin, then for another hour with puromycin and finally incubated 72 h with 100 nM gemcitabine in combination with anisomycin, puromycin or both. Cell death was measured by TMRM-staining, analyzed by flow cytometry. Data shown sum up three independent experiments and correspond to median with IQR as error bars. (**B**) Western blot analysis of KL, BiP and calreticulin abundance after 1 h anisomycin (200 nM) pretreatment and then 72 h incubation of IB115-empty vector and IB115 KL cells with 100 nM gemcitabine. Actin was used as loading control. To compare BiP and calreticulin abundance between conditions, results were all normalized to control condition (IB115-empty vector, no treatment). (**C**) Variations of relative cytosolic Ca^{2+} concentration were monitored by fluorescence video imaging in Fluo2-loaded IB115-KL cells bathed in Ca^{2+}-free HBSS medium, without any pretreatment (control, $n = 122$), or pretreated with gemcitabine at 100 nM, during 24 h ($n = 59$) or 48 h ($n = 66$). TG (10 nM) was added in the bath medium at 200 s to study reticular Ca^{2+}-leakage and 2 mM Ca^{2+} were applied at 600 s evaluate capacitive Ca^{2+} entry. In IB115-KL cells, the TG-releasable Ca^{2+} pool is increased after 24 h incubation with 100 nM gemcitabine but then, Ca^{2+} mobilization is reduced after 48 h treatment. Same trends are observed for capacitive Ca^{2+}-entry. This suggests that gemcitabine aggravates reticular Ca^{2+}-leakage in IB115-KL cells and leads to depletion of reticular Ca^{2+} pools quickly after a 48 h treatment. Data shown correspond to mean ± SD.

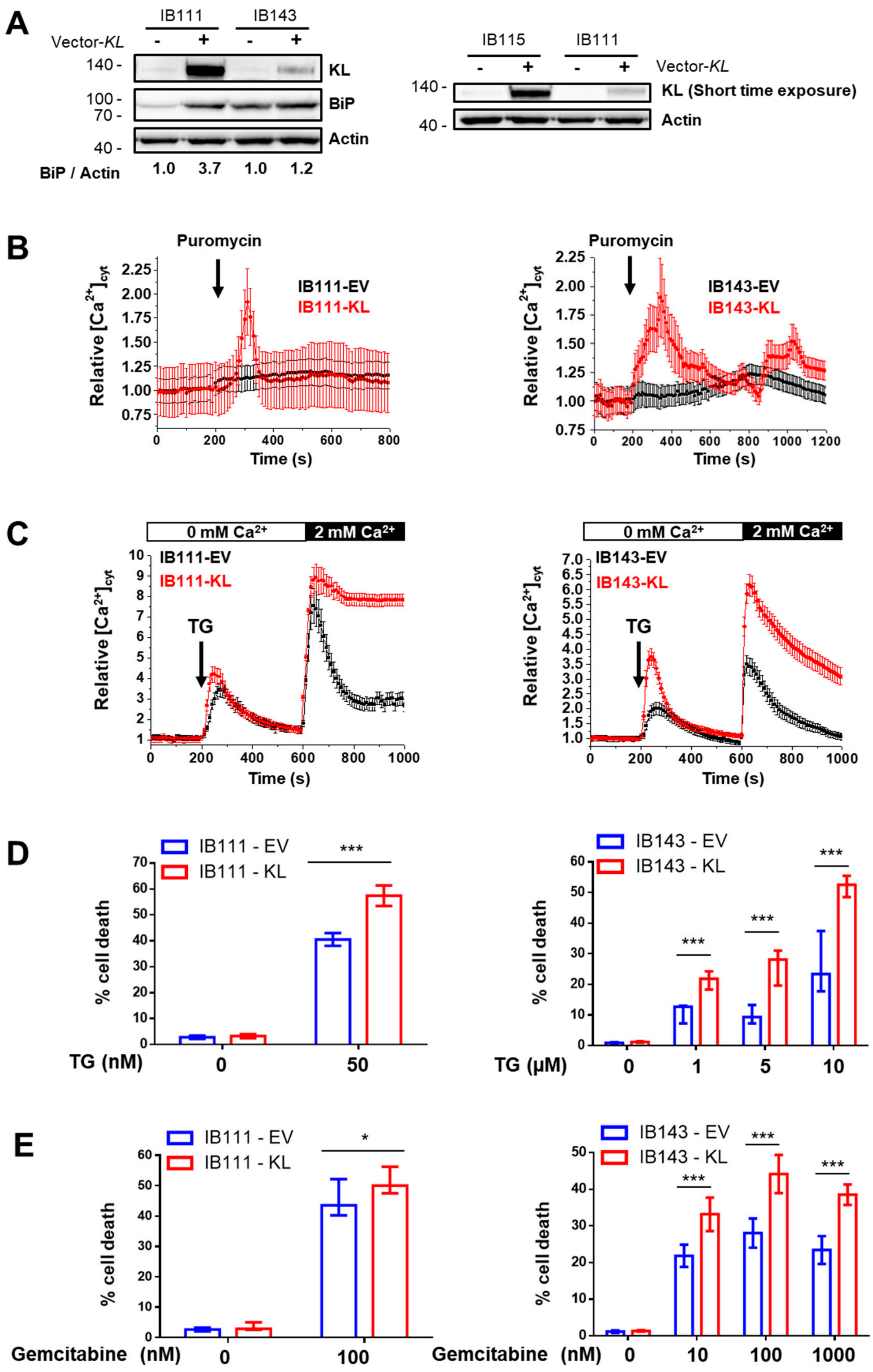

Figure S10. Sensitization to ER stressors by TLC opening was confirmed in two other DDLPS cell lines overexpressing *KL* (**A**) Western blot analysis of KL and BiP abundance in IB111 and IB143 cells overexpressing control vector (empty vector, EV) or *KL*. Actin was used as loading control. To compare BiP abundance, results were normalized to respective control set to 1. Despite a lower level

of *KL* expression compared to IB115-KL cells (see short time exposure on the right panel), BiP expression has increased more than 3-fold in IB111-KL cells compared to IB111-EV cell line and slightly in IB143-KL cells compared to IB143-EV. (**B**–**C**) Variations of relative cytosolic Ca^{2+} concentration were monitored, by fluorescence video imaging in Fluo2-loaded cells bathed in Ca^{2+}-free HBSS medium. Data are represented as mean ± SD and are representative of at least three independent experiments. (**B**) Puromycin (25 µg/mL) was added at 200 s to IB111-EV (n = 72), IB111-KL (n = 81) cells, IB143-EV (n = 35) and IB143-KL (n = 30) cells. Control cells do not react to puromycin addition but KL-overexpressing cells display a cytosolic Ca^{2+}-response, suggesting a constitutive reticular Ca^{2+}-leakage through the TLC in these cells, associated with increased BiP expression. (**C**) TG (10 nM) was added in the bath medium at 200 s to study reticular Ca^{2+}-leakage and 2 mM Ca^{2+} were applied at 600 s to evaluate capacitive Ca^{2+} entry. The increased cytosolic Ca^{2+}-response to TG and subsequent capacitive Ca^{2+}-entry in IB111-KL cells (n = 80) and IB143-KL cells (n = 40) compared to IB111-EV (n = 81) and IB143-EV (n = 40) cell lines, respectively, confirmed an enhanced reticular Ca^{2+}-leakage induced by Klotho. (**D**–**E**) Cell death was measured by TMRM-staining analyzed by flow cytometry. Histograms sum up three independent experiments with three replicates. (**D**) Cells were incubated for 72 h with TG at indicated concentrations. Results obtained with IB111 and IB143 cell lines are presented as mean ± SD and median ± IQR, respectively. We observed an increased sensitivity to cell death induced by TG (50 nM, 72 h), with on average 41% and 59% cell death in control and *KL*-overexpressing IB111 cell lines, respectively. Regarding the extremely resistant to ER stress IB143 cell line, Klotho still significantly increased death rate from 26% (as observed in control cells) to 52% after 72 h incubation with TG at 10 µM. (**E**) Gemcitabine treatment lasted 72 h for IB111 and 96 h for IB143 cell lines. Data shown are represented as median ± IQR for IB111 and as means ± SD for IB143 cell lines. At 100 nM, gemcitabine induced cell death on average in 45% of IB111-EV and in 51% of IB111-KL cells, and in 28% of IB143-EV and 44% of IB143-KL cells.

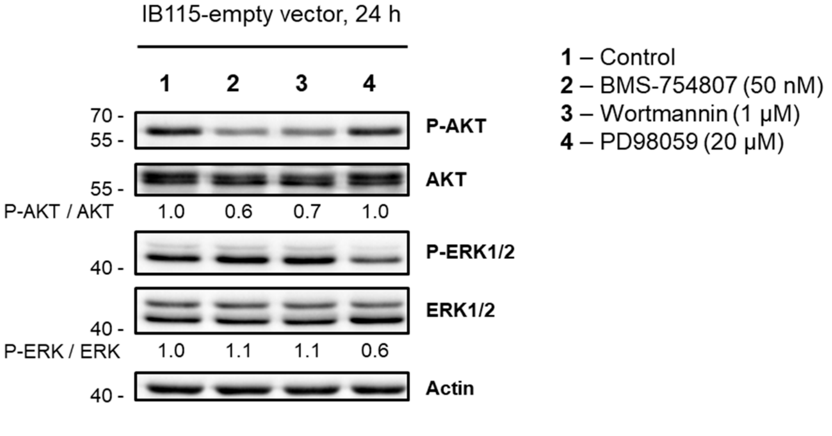

Figure S11. Western blot analysis of AKT and ERK1/2 phosphorylation status in IB115-empty vector cells after 24 h incubation with no drug, BMS-754807 (50 nM), Wortmannin (1 µM) or PD98059 (20 µM). Actin was used as loading control. For comparison of the ratios between phosphorylated form and total protein during treatments, results were all normalized to control condition (IB115-empty vector, no treatment). AKT phosphorylation was reduced by BMS-754807 and Wortmannin treatments but was not affected by PD98059, which efficiently and specifically reduced ERK1/2 phosphorylation level.

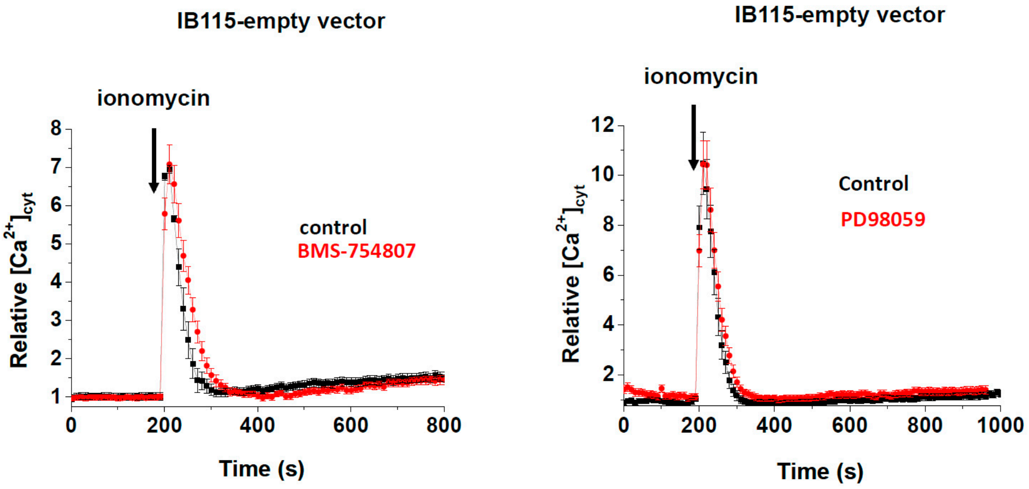

(**A**) (**B**)

Figure S12. Pretreatments with BMS-754807 or PD98059 does not affect reticular Ca^{2+} content. Variations of relative cytosolic Ca^{2+} concentration were monitored, by fluorescence video imaging in Fluo2-loaded IB115-empty vector cells bathed in Ca^{2+}-free HBSS, without any pretreatment (control, black line, n = 112), or pretreated with (**A**) BMS-754807 (50 nM, 24 h, n = 68) or (**B**) PD98059 (10 µM, 2 h, n = 78). Ionomycin (100 nM) was added in the bath medium at 200 s. Data shown correspond to mean ± SD.

Supplemental Material and Methods:

Cell Cycle Analysis

100 000 cells were seeded into 6-well plates in three replicates. The day after, culture medium was replaced by culture medium supplemented or not with gemcitabine at 10 nM, 100 nM or 100 µM. After 24, 48 or 72 h incubation, cells were trypsinized, washed with PBS 1X, fixed with ice-cold 70% ethanol and kept overnight at −20 °C. Then, cells were washed twice and stained with Propidium iodide with the kit FxCycle™ PI/RNase Staining Solution ((F10797, Life Technologies Inc., brand of ThermoFisher Scientific, Waltham, MA, USA)) according to the manufacturer's instructions. Samples were analyzed by flow cytometry (FACS Calibur, BD Biosciences, San Jose, CA, USA) using BD CellQuestPro software. Cell population was selected on a FSC/SSC dot plot. Doublets and clumps were excluded by gating out single cell population on a FL2W/FL2A dot plot diagram. For each condition, 10 000 single cells were analyzed. Cell cycle analysis was performed with FlowJo v10.1 (FlowJo LLC, Ashland, OR, USA) software. The proportion of cells in each cell cycle phase was determined thanks to a histogram showing DNA content distribution.

Apoptosis assay

To perform the assay, 50 000 cells were seeded into 12-well plates in three replicates. After 24 h, IB115 cell lines were pretreated or not 1 h with the caspase inhibitor QVD-OPh (20 µM) and then incubated 72 h with gemcitabine at 100 nM. Cell death was assessed by dual Annexin V-FITC/PI staining (Dead Cell Apoptosis Kit, Molecular Probes, Eugene, OR, USA) according to manufacturer's recommendations. For each condition, 10 000 cells were analyzed by flow cytometry (FACS Calibur, BD Biosciences, San Jose, CA, USA). Data were acquired using BD CellQuestPro software. Data analysis was performed with FlowJo v10.1 (FlowJo LLC, Ashland, OR, USA) software. Cell population was selected on a FSC/SSC dot plot. The percent of late apoptotic/necrotic cells was determined by gating out the Annexin V^+/PI^+ cell population on a FL1/FL2 dot plot diagram.

Gene expression analysis

IB115 (dedifferentiated liposarcoma) gene expression analysis was carried out using Agilent Whole human 44K Genome Oligo Array (Agilent Technologies, Santa Clara, CA, USA) according to the manufacturer's protocol [1] (Gene Expression Omnibus ID: GSE61791). Gene expressions were normalized within a batch of 39 tumors using the quantile method.

Reticular calcium imaging

Cells were transfected with Dharmafect Duo (Life Technologies Inc., brand of ThermoFisher Scientific, Waltham, MA, USA) 48 h prior to imaging. Few hours before transfection, 100 000 cells were plated per coverslip (24 mm dia.) placed within 6-well plates. Cells were transfected with a previously incubated transfection mix that contained per well: 2 µg of DNA plasmid for ErGAP1, 500 µL of serum free RPMI-1640/GlutaMAX-I (Life Technologies, Inc.) and 2 µL of Dharmafect Duo. The mix was incubated for 20 min at room temperature (RT), then cells were incubated with 500 µL of the transfection mix and 500 µL of serum free RPMI-1640/GlutaMAX-I overnight. The day after, transfection medium was replaced with complete medium for 24 h.

ErGAP1 is a genetic sensor based on the fusion of two jellyfish proteins: GFP and aequorin [2]. It specifically targets the endoplasmic reticulum (Kd = 16 µM). These experiments were performed at RT in calcium-free buffer (CFB) that consists of 40 mM NaCl (S9625), 5 mM KCl (P45041 mM MgCl2 (M2670), 10 mM HEPES (H3375) 10 mM glucose (G7528) and 1 mM EGTA, all from Sigma Aldrich (St. Quentin Fallavier, France).

Coverslips were mounted onto a magnetic chamber (Chamlide). The chamber was placed on a DMI6000 inverted wide-field microscope (Leica microsystem, Wetzlar, Germany). Imaging was acquired with an Orca-Flash 4.0 Scientific CMOS camera (Hamamatsu, Hamamatsu-city, Japan) using a 40x oil-immersion objective. Using a Lambda DG-4+ filter (Sutter instruments, Novato, CA, USA), ErGAP1 was excited at 403 and 470 nm and their respective emitted fluorescent lights were measured at 520 nm wavelength. Images (1024x1024 pixels) were taken with a 5 s time interval.

Mitochondrial calcium imaging

Single-cell mitochondrial calcium imaging was performed on Rhod2 and mitotracker-loaded cells with the same set-up as for cytosolic Ca^{2+} measurement. Cells were loaded with 3 µM Rhod2-AM and mitotracker green at room temperature in HBSS medium for 30 min and then incubated for 15 min in fluorescent probe-free HBSS to complete de-esterification of the dyes. Mitotracker green (excitation 485+/-22 nm, emission: 530+/-30 nm) was used to locate mitochondria and to draw regions of interest to restrict data collection to mitochondria. Mitochondrial Ca^{2+} changes were evaluated by exciting Rhod2-AM-loaded cells at 535+/-35 nm. The values of the emitted fluorescence (605+/-50 nm) for each cell (F) were normalized to the starting fluorescence (F0) and reported as F/F0 (relative $[Ca^{2+}]_{mito}$).

1. Lagarde, P.; Brulard, C.; Pérot, G.; Mauduit, O.; Delespaul, L.; Neuville, A.; Stoeckle, E.; Le Guellec, S.; Rochaix, P.; Coindre, J.M.; et al. Stable Instability of Sarcoma Cell Lines Genome Despite Intra-Tumoral Heterogeneity: A Genomic and Transcriptomic Study of Sarcoma Cell Lines. *Austin J. Genet. Genomic. Res.* **2015**, *2*, 1014
2. Rodriguez-Garcia, A.; Rojo-Ruiz, J.; Navas-Navarro, P.; Aulestia, F.J.; Gallego-Sandin, S.; Garcia-Sancho, J.; Alonso, M.T. GAP, an aequorin-based fluorescent indicator for imaging Ca2+ in organelles. *Proc. Natl. Acad. Sci. USA.* **2014**, 111, 2584–2589.

© 2018 by the authors. Licensee MDPI, Basel, Switzerland. This article is an open access article distributed under the terms and conditions of the Creative Commons Attribution (CC BY) license (http://creativecommons.org/licenses/by/4.0/).
